# Catalysis of a Diels–Alder Reaction between Azachalcones and Cyclopentadiene by a Recyclable Copper(II)-PEIP Metal-Organic Framework

**DOI:** 10.3390/ma16155298

**Published:** 2023-07-27

**Authors:** Eleni Hadjikyprianou, Sotiris Petrides, Andreas Kourtellaris, Anastasios J. Tasiopoulos, Savvas N. Georgiades

**Affiliations:** Department of Chemistry, University of Cyprus, 1 Panepistimiou Avenue, Aglandjia, 2109 Nicosia, Cyprus

**Keywords:** MOF, Diels–Alder cycloaddition, heterogeneous catalysis, green solvent, sustainability

## Abstract

Metal-organic frameworks (MOFs) have attracted considerable interest as emerging heterogeneous catalysts for organic transformations of synthetic utility. Herein, a Lewis-acidic MOF, {[Cu_3_(PEIP)_2_(5-NH_2_-*m*BDC)(DMF)]·7DMF}∞, denoted as Cu(ΙΙ)-PEIP, has been synthesized via a one-pot process and deployed as an efficient heterogeneous catalyst for a Diels–Alder cycloaddition. Specifically, the [4 + 2] cycloaddition of 13 substituted azachalcone dienophiles with cyclopentadiene has been investigated. MOF-catalyzed reaction conditions were optimized, leading to the selection of water as the solvent, in the presence of 10% mol sodium dodecyl sulfate (SDS) to address substrate solubility. The Cu(II)-PEIP catalyst showed excellent activity under these green and mild conditions, exhibiting comparable or, in some cases, superior efficiency to a homogeneous catalyst often employed in Diels–Alder reactions, namely, Cu(OTf)_2_. The nature of the azachalcone substituent played a significant role in the reactivity of the dienophiles, with electron-withdrawing (EW) substituents enhancing conversion and electron-donating (ED) ones exhibiting the opposite effect. Coordinating substituents appeared to enhance the endo selectivity. Importantly, the Cu(II)-PEIP catalyst can be readily isolated from the reaction mixture and recycled up to four times without any significant reduction in conversion or selectivity.

## 1. Introduction

Metal–organic frameworks (MOFs) have attracted significant research attention over the last few decades due to their highly porous structures [1] and large surface areas [2,3], as well as the presence of metal cations that may exhibit interesting physical properties and/or act as Lewis acidic sites [4]. As anticipated, MOFs have been proven to be versatile platforms for applications in diverse areas, including gas storage and separation [5,6,7], magnetism [8], sensing [9,10], and catalysis [11,12]. During the last few years, there has been a growing interest on the use of MOFs in catalysis due to their unique features, including: (i) their crystalline nature and high-yield synthesis from low-cost starting materials; (ii) their hybrid organic-inorganic nature and the existence of both Lewis acidic and basic sites in their structures [12]; (iii) their extraordinary structural and architectural diversity and the variety of pore shapes and dimensions that render them ideal hosts for a series of substrates; (iv) the capability to modify/tailor their structures [13] to achieve selected applications; and (v) their chemical and thermal stability that allows their reactivation and reuse for multiple catalytic cycles [14].

For these reasons, MOFs, among various other types of nanomaterials [15,16], have been examined as heterogeneous catalysts and have shown very promising results for several organic transformations, including Knoevenagel condensations [17,18], Friedel-Crafts [19,20], C-N cross-coupling [21], and ‘click’ reactions [22,23]. Another type of reaction that has been explored using MOF catalysts is the Diels–Alder (D-A) cycloaddition, however, there are still limited studies in this area [24,25,26,27,28,29,30]. In particular, Cu(II), Cr(III), Fe(III), Zr(IV), and mixed Fe(II)/Ni(II)-based MOFs with various ligands and structural architectures have been examined as D-A catalysts for a series of diene and dienophile substrate combinations, exhibiting mixed results in terms of conversion and selectivity. Notably, previous studies employing MOFs have not investigated the formation of bridged bicyclo[2.2.1]hept-2-ene-type D-A adducts, while many of them employed high boiling point organic solvents and/or required high reaction temperatures [24,28,29].

The [4 + 2] cycloaddition, commonly known as the Diels–Alder reaction [31,32], is one of the most emblematic pericyclic reactions in organic chemistry and, deservedly, one of the most frequently employed in organic synthesis, due to its synthetic reliability and atom-economic nature for the facile construction of complex 6-membered ring systems, most notably (fused) cyclic scaffolds of natural products [33,34]. In many cases, the reaction requires elevated temperature, organic solvent, and a Lewis-acidic catalyst [35]. Generation of valuable D-A adducts via MOF catalysis would bypass time- and resource-consuming purification steps that are needed after homogeneous catalysis to separate the product from the catalyst.

In the present work, a MOF material previously reported by one of our group [36], referred to as Cu(II)-PEIP, is assessed for the first time as a heterogeneous catalyst. This MOF formed in a simple high-yield, one-pot reaction of a Cu(II) salt (Cu(NO_3_)_2_·2.5H_2_O) and a Schiff base ligand generated in situ from the condensation of 5-amino-isophthalic acid (5-NH_2_-*m*BDCH_2_) and 4-pyridine-carboxaldehyde (4-PyrCA). Cu(II)-PEIP is a microporous 3D MOF containing coordinatively unsaturated Cu(II) sites, multiple N-donor atoms, and free -NH_2_ groups. Based on previous findings [36], Cu(II)-PEIP displays an appreciable BET area of 1785 m^2^.g^−1^ and demonstrates high CO_2_ uptake (reaching 4.75 mmol.g^−1^ at 273 K and 1 bar) and good CO_2_/CH_4_ selectivity (8.5 at zero coverage and 273 K). Interestingly, these values are higher than the corresponding ones of other high-surface area MOFs, including MOF-5, MOF-200 and MOF-210. The high porosity of Cu(II)-PEIP, in combination with the presence of unsaturated Cu(II) sites and the existence of multiple functional groups, which are potentially useful for interaction with substrates, render this MOF a suitable candidate for Lewis acid catalysis. For these reasons, Cu(II)-PEIP was chosen to be investigated as a heterogeneous catalyst for a [4 + 2] cycloaddition reaction.

Herein, we report the catalytic activity of the Cu(II)-PEIP material for a D-A cycloaddition between a family of azachalcones (i.e., substituted (pyridinyl)propenones) and cyclopentadiene, to generate synthetically valuable bicyclo[2.2.1]hept-2-ene products. The building units investigated were selected mainly on the basis of their capability to coordinate the MOF’s metal centers as well as be involved in hydrogen bond-type interactions. This mild, room-temperature catalytic process has been optimized by selecting a green, non-toxic solvent (water) and achieves near-quantitative transformation for most of the substrates, in the presence of a surfactant (sodium dodecyl sulfate, SDS). Cu(II)-PEIP displays excellent activity under these conditions at low catalyst loading (2% mol), and can be recycled up to four times, as indicated by powder X-ray diffraction (PXRD) studies, indicating the potential of this material for use in sustainable processes.

## 2. Materials and Methods

### 2.1. General

Reagent grade chemicals (purity > 97%) were obtained from commercial sources (Sigma-Aldrich (St. Louis, MO, USA), Alfa Aesar (Haverhill, MA, USA), TCI Europe N.V. (Zwijndrecht, Belgium) and used without further purification. Millipore (double-distilled) water was in-house prepared. Organic solvents used for reactions, obtained from Carlo Erba Reagents (Milano, Italy), were anhydrous, unless otherwise stated. Organic solvents used for chromatography, obtained from Merck (Burlington, MA, USA), were of analytical grade. All synthetic procedures were conducted under air, unless otherwise specified.

Flash chromatographic purifications were performed in glass columns, using silica gel 60 (0.063–0.2 mm) from Merck as stationary phase. Thin layer chromatography (TLC), to monitor reaction progress, was performed on aluminum plates covered with silica gel 60 (F254) from Merck, which allowed compound visualization under a UV lamp (254 nm) as dark spots on a green background. TLC staining was performed with vanillin (TCI Europe N.V.) where necessary.

Nuclear magnetic resonance (NMR) spectra were obtained on a Bruker Avance III 500 Ultrashield Plus spectrometer (at 500 MHz for ^1^H NMR and 125 MHz for ^13^C NMR, at 25 °C). Chemical shift calibration was based on the NMR solvent’s residual peak. Deuterated solvents were obtained from Merck or TCI Europe N.V.

Powder X-ray diffraction (PXRD) was performed on a RIGAKU Miniflex 6th g diffractometer (Neu-Isenburg, Germany), using Cu-Kα radiation (λ = 1.5406 Å).

### 2.2. Cu(II)-PEIP MOF Preparation

**Figure 1:**
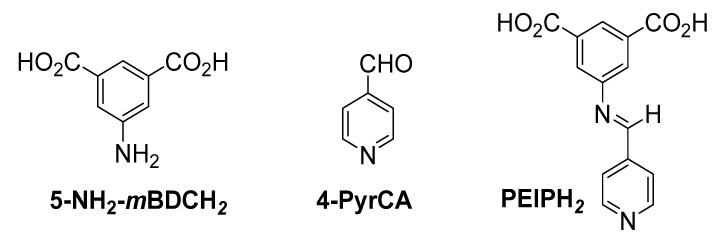
Structures of constituent components and in situ generated ligand (PEIPH_2_) employed in the synthesis of Cu(II)-PEIP MOF.

The Cu(II)-PEIP MOF was prepared by means of a variation of the previously described protocol [36], which involved in situ synthesis of the PEIPH*_2_* ligand via condensation of 5-amino-isophthalic acid (5-NH_2_-*m*BDCH_2_) and 4-pyridinecarboxaldehyde (4-PyrCA) (see structures in Figure 1). The two components, 5-NH_2_-*m*BDCH_2_ (0.08 g, 0.44 mmol) and 4-PyrCA (43 μL, 0.45 mmol), were dissolved in 5 mL of *N*,*N*-dimethylformamide (DMF) in a 20 mL screw-cap vial. After 5 min of sonication, solid Cu(NO_3_)_2_·2.5H_2_O (0.08 g, 0.34 mmol) was added to the solution. The mixture was sonicated for 5 more minutes and then heated, without stirring, at 100 °C for 24 h. During this period, bright green polyhedral crystals of Cu(II)-PEIP were formed. The crystals were thoroughly washed with DMF to remove unreacted starting materials from the pores of the MOF. Subsequently, they were washed several times with acetone and dried under vacuum. The yield was approximately 70%. Powder X-ray diffraction (PXRD) was used to confirm the purity and crystallinity of the batch product. Digestion of a representative sample of the MOF with HCl and subsequent ^1^H NMR analysis confirmed the complete removal of DMF from the pores of Cu(II)-PEIP MOF (Figure S2, Supporting Information).

### 2.3. Synthesis of Azachalcone Dienophile Building Units

Thirteen (13) azachalcone dienophiles with various substituents (**1a–m**, Scheme S1, Supporting Information) were synthesized via aldol reaction of the corresponding aromatic aldehydes with an enolate generated from 2-acetylpyridine under basic conditions, followed by in situ dehydration. For this transformation, various literature protocols were applied, depending on aldehyde substrate reactivity [37,38,39,40] (see Table S1, Supporting Information). The azachalcone products were isolated via filtration or via chromatography, depending on product solubility in the reaction solvent, and characterized by ^1^H and ^13^C NMR prior to use in the D-A reaction (>95% purity).

### 2.4. Screening of Conditions for [4 + 2] Cycloaddition (Diels–Alder Reaction)

**Scheme 1:**
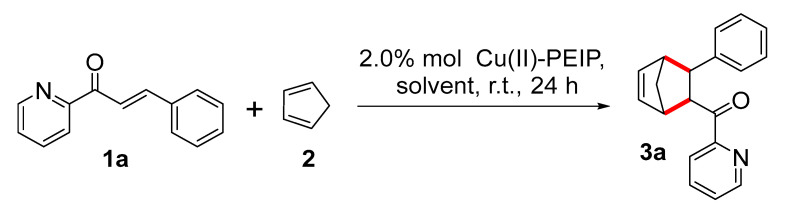
Model Diels–Alder cycloaddition employed for the evaluation of various solvent systems.

Various solvent systems were tested prior to selecting an optimal one for the MOF-catalyzed D-A reaction on a model system involving the mother (unsubstituted) azachalcone structure (**1a**, 0.25 mmol) and freshly-distilled cyclopentadiene (**2**, 2.5 mmol) in 1 mL of solvent (Scheme 1). The Cu(II) equivalents from the MOF in all trials were kept at 2% mol (relative to **1a**) and the reactions were carried out with gentle stirring on a combi-chem fixed-speed Stuart SB2 rotator (Cole-Parmer UK, Cambridgeshire, UK) to achieve reproducible mixing and avoid MOF attrition and degradation. Solvents which were found useful in previous catalytic studies with MOF materials were included in this screening. Tested solvents were acetonitrile (CH_3_CN), dichloromethane (DCM), tert-butyl methyl ether (BME), *N*-methylpyrrolidone (NMP), *N*,*N*-dimethylformamide (DMF), methanol (MeOH), ethanol (EtOH), and water (H_2_O), while aqueous systems containing adducts (NaCl, sodium dodecyl sulfate-SDS) were also tested on the model system, after it become obvious that some of the substituted azachalcone substrates required an adduct in order to become fully soluble in water. For optimization, we required high to quantitative reaction conversion (>95%), as well as the ability of the MOF to remain insoluble (>95% recovery) in the solvent, in order to serve as a heterogeneous catalyst (results shown in Table 1). Conversion was assessed based on product isolation, after reaction on the model system was allowed to proceed for 24 h at room temperature. Determination of the insoluble MOF mass was conducted by recovering the MOF after the reaction via filtration, washing with diethyl ether and acetone, and drying. The selected conditions were then applied in the MOF-catalyzed D-A reactions on all azachalcone substrates (see Section 2.6).

### 2.5. Control Experiments for Assessing the Effect of Individual MOF Components in Catalysis and Control Reactions with a Homogeneous Copper(II) Catalyst, Cu(OTf)_2_

Once appropriate reaction conditions had been selected, as described in Section 2.4, a number of control experiments were conducted on the model substrate combination **1a** (unsubstituted azachalcone, 0.25 mmol) + **2** (cyclopentadiene, 2.5 mmol), under comparable reaction conditions to the optimal ones (1 mL of solvent, r.t., 24 h), to detect any catalytic effect of each of the individual MOF components/ligands (5-NH_2_-*m*BDCH_2_ and PEIPH_2_) as well as the free copper salt (Cu(NO_3_)_2_·2.5H_2_O) employed in the self-assembly process of MOF formation (results in Table 2). Products were isolated after extraction to organic solvent and column chromatography.

Additionally, control D-A reactions, promoted by a homogeneous catalyst (5% mol Cu(II)), were conducted for all actual azachalcone substrates (dienophiles) included in this study (**1a–m,** 0.25 mmol) in combination with diene **2** (2.5 mmol) in 1 mL of solvent. Specifically, the Lewis-acidic catalyst Cu(OTf)_2_, a standard Diels–Alder catalyst, was employed in order to have a comparison to the heterogeneous D-A reactions catalyzed by the Cu(II)-PEIP MOF material (results discussed in Section 3.4). Products were isolated after extraction to EtOAc and column chromatography.

### 2.6. Procedure for Cu(II)-PEIP MOF-Catalyzed [4 + 2] (Diels–Alder) Cycloaddition

All D-A reactions were carried out in parallel format at least 3 times each, to check reproducibility, in 1.5 mL Eppendorf tube scale. To a mixture containing the Cu(II)-PEIP MOF (3 mg, 2% mol Cu(II) loading) in water (1 mL), we added SDS (0.0072 g, 0.025 mmol, 0.1 equiv.), and the mixture was agitated gently by hand to create a fine suspension. Subsequently, an azachalcone dienophile (0.25 mmol, 1 equiv.) was added, followed by an excess of freshly-distilled cyclopentadiene (**2**, 2.5 mmol, 10 equiv.). The resulting mixtures were rotated at fixed speed at room temperature on a combi-chem Stuart SB2 rotator for 18 h. The heterogeneous catalyst (MOF) was subsequently filtered and washed with distilled water, diethyl ether, and acetone. The aqueous filtrate (and wash solvent) was transferred to a separatory funnel and extracted with EtOAc (x3). The combined organic phase was washed with brine and dried over anhydrous Na_2_SO_4_. After removal of the drying agent, the crude mixture was exhaustively dried on the rotary evaporator, weighted, and characterized by ^1^H NMR to determine conversion and endo:exo product molar ratio. For most cases, NMR revealed the absence of any organic impurities. In one case, which was not quantitative, the D-A adduct (**3i**) was separated from the unreacted dienophile precursor (**1i**) after column chromatography, and its purity was confirmed by ^1^H NMR. Conversions and endo:exo ratios are discussed in Section 3.4.

### 2.7. Assessment of MOF’s Structural Integrity under D-A Conditions, and after each Catalytic Cycle

Powder X-ray diffraction (PXRD) was employed as a method of choice to assess the structural integrity of the Cu(II)-PEIP MOF material. Initially, a control experiment was conducted where the MOF was submitted to the optimal D-A conditions for various times, in the absence of any substrate, then recovered, washed (distilled water, diethyl ether, acetone), dried and examined by PXRD, to provide an idea of whether it maintained its original structure (see Figure S3, Supporting Information).

Similarly, PXRD on the recovered MOF after each round of D-A catalysis on the model substrates system (**1a** + **2**), considered to be representative of all the substrates tested, was employed to determine the number of cycles allowed before the MOF begins to show signs of deterioration (results discussed in Section 3.5).

## 3. Results and Discussion

### 3.1. Suitability of Cu(II)-PEIP MOF for Catalysis

**Figure 2:**
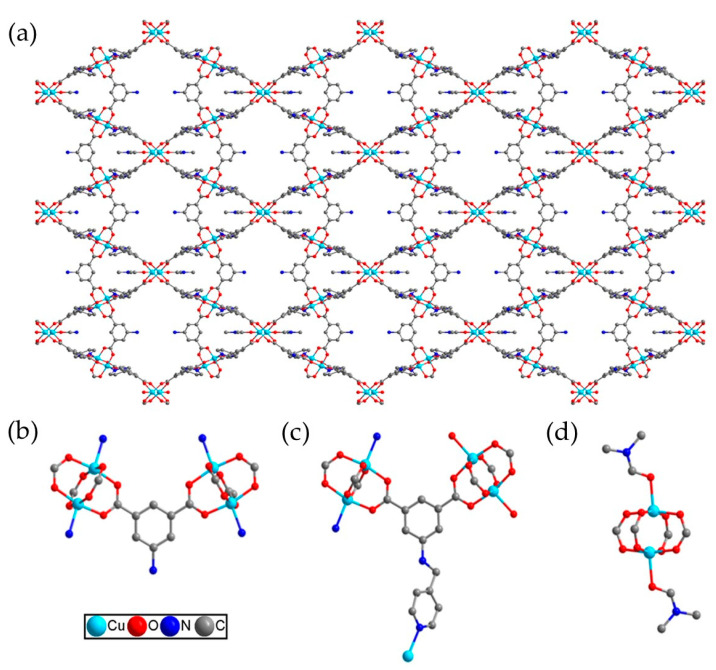
(**a**) Representation of the 3D Cu(II)-PEIP MOF material along the *a* axis. (**b**,**c**) Coordination modes of 5-NH_2_-*m*BDC^2−^ and PEIP^2−^ ligands, respectively. (**d**) Dinuclear paddle-wheel secondary building unit [Cu_2_(RCOO)_4_(DMF)_2_], believed to be the catalytic site. [H atoms are omitted for clarity].

The MOF, Cu(II)-PEIP, was prepared by a slight variation of a method in the previous literature [36], which involved in situ formation (via condensation) of an imine ligand (PEIPH_2_) from aniline 5-NH_2_-*m*BDCH_2_ and aldehyde 4-PyrCA, and the subsequent self-assembly of the MOF. The synthesized MOF (Figure 2a) comprises both the 5-NH_2_-*m*BDC^2−^ and PEIP^2−^ ligands connecting four and five Cu(II) ions, respectively (Figure 2b,c), giving rise to a 3D structure.

The 3D pores of this material are of appropriate dimensions [36] to accommodate substrates and bridged D-A adducts, such as the ones included in the current study, while the availability of Lewis-acidic Cu(II) centers was deemed promising for D-A catalysis. An exhaustive wash of the generated MOF material with a volatile, weakly coordinating solvent, such as acetone, ensured that excess/uncoordinated reaction solvent (DMF), trapped in the pores during MOF synthesis, was efficiently removed to free up space. Small amounts of DMF, detected by ^1^H NMR in the washed material after acidic digestion (see Figure S2, Supporting Information), can be attributed to copper-coordinated DMF molecules, which are also evident in the crystal structure (Figure 2d).

The presence of dinuclear paddle-wheel secondary building units, [Cu_2_(RCOO)_4_(DMF)_2_] within the structure of Cu(II)-PEIP (Figure 2d) is of great interest for this specific application due to the fact that solvent molecules (DMF) are weakly coordinated to the metal centers (see Figure S1a, Supporting Information) and can be readily exchanged upon contact with (Lewis-basic) substrates capable of coordination, such as the azachalcone dienophiles employed in this study. The latter are present in the reaction in excess relative to the copper(II) sites. Therefore, the paddle-wheel copper(II) sites contribute the expected catalytic centers of the Cu(II)-PEIP MOF material for the D-A reaction. The presence of free aniline NH_2_ groups, in proximity to the Cu(II) sites (see Figure S1b, Supporting Information), could potentially contribute to the positioning of dienophiles.

The aforementioned features of this MOF led to its selection for the catalysis of D-A reactions between azachalcones and cyclopentadiene. It is worth mentioning that it is the first time that the formation of bridgehead D-A cycloadducts exhibiting a 3D structural element has been reported by means of MOF catalysis, which is allowed due to the large pore size of the Cu(II)-PEIP catalyst.

### 3.2. Selection of Reaction Solvent for D-A Cycloaddition

Investigations on the effect of the solvent, as the most important parameter for optimization of the D-A cycloaddition, were carried out in the presence of MOF at 2% mol of Cu(II). The purpose of the solvent screening was two-fold: (i) to identify a solvent in which the MOF maintains its structural integrity (i.e., exhibits lack of solubility, based on its recovered mass), and (ii) to ensure that, in the selected solvent, the model dienophile **1a** is fully converted to the desired D-A adduct (**3a**) in the presence of excess diene (cyclopentadiene, **2**). All other parameters (reaction scale, substrate concentration, temperature, and reaction time) were kept fixed.

In all solvent systems tested on the model substrates combination **1a** + **2** (Scheme 1), the Cu(II)-PEIP MOF remained insoluble to the desired extent (>95% based on mass recovery), while conversions were significantly higher in aqueous solvents (based on isolated product mass and ^1^H NMR analysis) (Table 1), possibly due to a contribution from the hydrophobic effect. Focusing on the need to select an environmentally-benign solvent system, we selected an aqueous system for the actual D-A reactions, which also appeared to afford near-quantitative conversion (~99%). In the process of testing the substituted azachalcone dienophiles, it was found that some of them (specifically the more hydrophobic ones) required the addition of a surfactant, sodium dodecylsulfate (SDS), in order to become entirely soluble in water, and, therefore, SDS modified conditions were added to the original screening set. Eventually, we selected water with 10% mol SDS as the ideal conditions for the application of the heterogeneous Cu(II)-PEIP catalyst in D-A reactions.

It is noted that the stability of the MOF catalyst, after 2–72 h agitation in the selected solvent system, in the absence of any substrate was confirmed by examining the isolated MOF by powder X-ray diffraction (PXRD). The PXRD pattern suggested no significant structural deterioration (Figure S3, Supporting Information), and, therefore, the selected solvent was considered compatible with the MOF. This was also confirmed from the ^1^H NMR spectra of the supernatant liquid of MOF material treated in the experiment’s aqueous conditions (D_2_O/10% mol SDS) for 24 h, 48 h, and 72 h, which indicated the absence of leaching of the organic ligands (Figure S4, Supporting Information).

### 3.3. Ability of Individual Components of Cu(II)-PEIP MOF to Catalyze a Model D-A Reaction

A series of control D-A reactions, assessing the conversion of model substrates **1a** + **2**, were performed in solution in the selected solvent system (H_2_O containing 10% mol SDS) and under similar conditions as in the optimization (r.t., 24 h). These control reactions were carried out in the absence of Cu(II)-PEIP MOF but in the presence of either Cu(II) cations or one of the MOF’s constituent ligands (PEIPH_2_ or 5-NH_2_-*m*BDCH_2_) in their neutral form (Table 2). The additive-free D-A reaction, involving reactants only, was also studied. The purpose of these control experiments was to determine whether the entire MOF or its individual components serve as the catalyst in D-A, or if a catalyst is required at all.

As evident from the results, summarized in Table 2, the individual components, as well as the additive-free (uncatalyzed) reaction, all afforded low conversions to the D-A adduct (**3a**). Among these, Cu(II) cations from Cu(NO_3_)_2_·2.5H_2_O (Entry 1) afforded the highest conversion (35%) in the same aqueous system selected for the MOF-catalyzed reactions, albeit with no observed diastereoselectivity (endo:exo = 1:1). In the case of the free ligands (Brønsted-acidic), even lower yields were obtained (Entry 2: PEIPH_2_, 25%; Entry 3: 5-NH_2_-*m*BDCH_2_, 29%), with a similar lack of diastereoselectivity. Finally, in the uncatalyzed case (Entry 4), only trace product was observed (<2%). This confirms that the MOF in its entity is the main Lewis acid catalyst in the MOF-containing reaction systems (see Section 3.4).

### 3.4. Investigation of Azachalcone Dienophile Scope in Cu(II)-PEIP-Catalyzed D-A

**Scheme 2:**
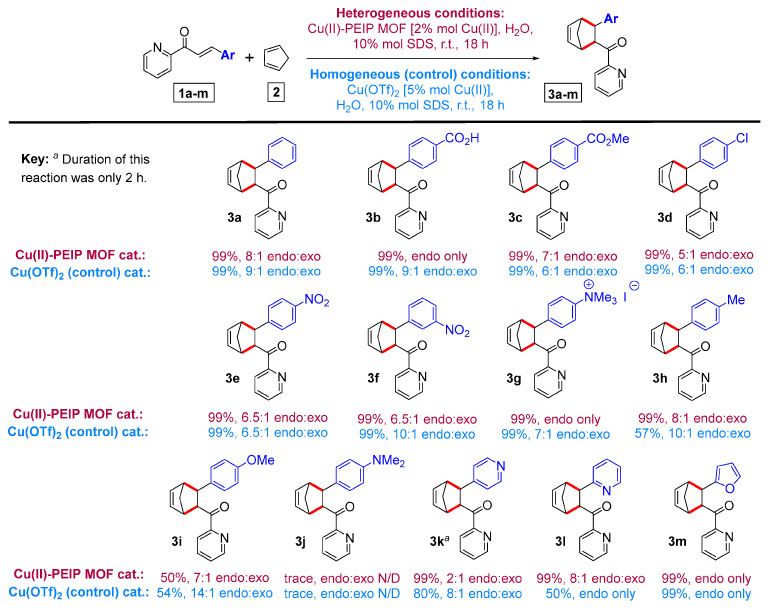
Parallel Diels–Alder reactions of azachalcone dienophiles **1a–m** (1 equiv.) with diene **2** (10 equiv.), catalyzed by Cu(II)-PEIP MOF. A representative set of electron-deficient and electron-rich dienophiles were included to assess the scope of the MOF-catalyzed D-A (data in red). Control reactions were performed in solution, with homogeneous catalyst Cu(OTf)_2_ for comparison (data in blue). Inset: Structures of obtained products (major = endo D-A adduct, racemic), reaction conversions, and endo:exo molar ratios, as determined by ^1^H NMR. The Cu(II)-PEIP affords excellent conversions (~99%) for most substrates and remarkable endo selectivities, rivaling those of the homogeneous catalyst.

After the optimal conditions of solvent and temperature were determined, and after adopting a shorter than 24 h but sufficient reaction duration (18 h), the selected protocol was applied on the full range of thirteen (13) azachalcone dienophile substrates (**1a–m**), always using cyclopentadiene (**2**) as the diene, for parallel Cu(II)-PEIP-catalyzed D-A reactions (Scheme 2). Some of the dienophiles comprised a phenyl ring, either unsubstituted (**1a**) or monosubstituted with a range of chemical functionalities (**1b–j**), while in others the phenyl ring was substituted for a pyridine (**1k,l**) or furan ring (**1m**). This range of substrates provided an opportunity to study the electron-withdrawing and the electron-donating effects on D-A reaction conversion and selectivity.

After completion of a typical D-A reaction, the MOF material could be readily separated and recovered by filtration, while the supernatant was extracted to ethyl acetate (to remove SDS), exhaustively dried (to remove excess cyclopentadiene, **2**), and analyzed directly by ^1^H NMR spectroscopy without a need for further purification, since most substrates afforded >98% conversion (except for the case of substrate **1i**, where chromatography was needed; and **1j**, where no product was observed).Meticulous ^1^H and ^13^C NMR analysis ensured structural characterization of each isolated product (for the data, see Section S1 of Supporting Information). The molar ratio of endo-to-exo D-A adducts obtained from each substrate was determined by relative peak integration in ^1^H NMR, while conversion was calculated based on the mass of isolated organic material. With the exception of **1i** and **1j** reaction crudes, which contained azachalcone starting material, the rest of the products did not contain any detectable organic impurity. The conversions and endo-to-exo molar ratios under Cu(II)-PEIP catalysis (heterogeneous conditions) are summarized in Scheme 2.

The diverse set of substrates employed in this study demonstrated a dual behavior in the D-A reaction catalyzed by the Cu(II)-PEIP MOF. The reaction afforded quantitative conversions with the electron-deficient 4-carboxy- (**1b**), 4-methylester- (**1c**), 4-chloro- (**1d**), 4-nitro- (**1e**), 3-nitro- (**1f**), and 4-trimethylammonium-phenyl (**1g**) azachalcone substrates, as well as with the 4- and 2-pyridyl ones (**1k** and **1l**, respectively), revealing an excellent efficiency of the MOF catalyst when used in combination with a range of electron acceptor-substituted dienophiles. The behaviour of this dienophile sub-set is consistent with predictions of Fukui’s Frontier Molecular Orbital (FMO) theory, which correlates the success of normal electron demand Diels–Alder reactions with a small FMO gap between the energies of the diene’s HOMO and the dienophile’s LUMO [41]. Electron-withdrawing groups on the dienophile lower the dienophile’s LUMO energy level, leading to a small FMO gap and facilitating the reaction. The unsubstituted substrate (**1a**), the weak electron donor-substituted 4-methyl-phenyl (**1h**), and especially the π-extended 2-furyl one (**1m**) gratifyingly exhibited similar quantitative conversion to the corresponding D-A adducts, suggesting a permissive FMO gap. Moderate or poor catalytic efficiency was observed only for two strongly electron-rich dienophiles, namely, the 4-methoxy-phenyl (**1i**, 50% conversion) and 4-dimethylamino-phenyl (**1j**, no conversion). This was not surprising, considering the contribution of the electron donor in raising the dienophile’s LUMO energy level, resulting in a mismatch with the diene’s HOMO, which manifests as poor conversion.

Importantly, the heterogeneous Cu(II)-PEIP catalyst (at 2% mol Cu(II) loading) afforded, in almost all of the studied cases, comparable or even higher conversions compared to a (standard for D-A) homogeneous Lewis-acidic catalyst (Cu(OTf)_2_), employed herein as a control catalytic system, at 5% mol Cu(II) in the same solvent (H_2_O, containing 10% mol SDS) and with same reaction duration (18 h). Conversions and endo-to-exo results with Cu(OTf)_2_ (homogeneous control conditions) are also included in Scheme 2 for comparison with the MOF case.

Another interesting finding was a significant difference in endo-to-exo selectivity observed between dienophiles with different electronic features. In particular, dienophiles bearing an additional Lewis-basic moiety with coordinating ability, such as **1b** (CO_2_H in anionic form) and **1m** (2-furyl), exhibited the most enhanced endo selectivity, manifested as an absence of the exo D-A adduct. Following a similar trend, substrate **1l** (2-pyridyl) demonstrated a higher endo-to-exo selectivity (8:1) compared to other substrates without coordinating ability. In these cases, the coordinating moiety could potentially promote an additional interaction to a Cu(II) center within the MOF, thus affecting substrate orientation in the transition state and playing an active role in controlling diastereoselectivity in favor of the endo adduct. To gain further insight into the effect of coordinating moieties, dienophile **1c** was included in the study, in which the carboxy-moiety was protected to a methyl ester (CO_2_Me). As expected, the endo-to-exo selectivity declined to 7:1, which may be indicative of the coordinating role of the carboxy-anion in related substrate **1b**. Substrate **1k** (4-pyridyl) appears to violate this observed trend. This may be attributed to the misplacement of the nitrogen atom, which in the pyridine’s 4-position is unable to synergize with the azachalcone’s carbonyl or 2-pyridine when it comes to metal center coordination. Finally, the unusual trimethylammonium-phenyl dienophile (**1g**) also exclusively afforded the endo product (**3g**), implying a role of electrostatic or steric repulsions with MOF components, or alternatively a potential association with the MOF catalyst’s Lewis-basic residues via cation-dipole interaction. However, the study of an extended series of analogues is necessary, supported by computational methods, to draw more systematic conclusions, and this is planned at a later stage.

It is noteworthy that the endo selectivity results obtained with Cu(II)-PEIP, especially in the cases of **3b** (CO_2_H), **3g** (NMe_3_^+^), and **3m** (2-furyl), where the endo is the exclusive product, rivaled those obtained with the homogeneous catalyst Cu(OTf)_2_ (Scheme 2). In some other cases, the two catalytic systems were comparable.

### 3.5. Recyclability of Cu(II)-PEIP Catalyst

**Figure 3:**
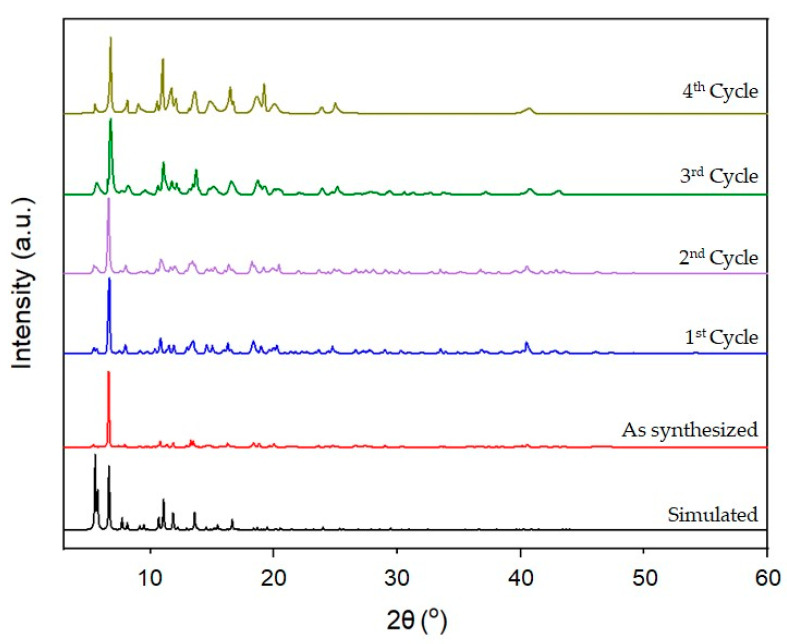
Comparison of the powder X-ray diffraction (PXRD) pattern of the pristine Cu(II)-PEIP catalyst (red) with the simulated from single crystal X-ray diffraction data pattern, and with the ones from each of the first four D-A reaction cycles.

One of the potential advantages of using MOFs as heterogeneous catalysts is their recyclability. The Cu(II)-PEIP MOF that was recovered after the first D-A reaction on model substrates **1a** + **2** was re-submitted to new reaction several times (will be referred to as cycles) in order to check whether it retains its ability to catalyze the reaction. By visual inspection, it was found that the external appearance of Cu(II)-PEIP after each of the first four reaction cycles was unchanged, which was an indication of crystallinity retention. The conversion of substrates to D-A adduct in the first four reaction cycles appeared to remain high (>95%), while only after the fifth cycle did it start to diminish. The retention of the crystallinity and the structural integrity of the recycled Cu(II)-PEIP MOF was also supported by the PXRD patterns of the MOF material after each of the first four catalytic cycles (Figure 3).

Furthermore, different batches of the model MOF-catalyzed D-A reaction (**1a** + **2**), using Cu(II)-PEIP catalyst that was treated under the selected aqueous conditions for up to 72 h prior to the actual D-A reaction, revealed reproducible and quantitative conversion of model dienophile **1a** to the corresponding product **3a,** independently of the time of the pre-treatment of the MOF (Figure S5, Supporting Information). The obtained ^1^H NMR spectra are indicative of both product purity and the retention of the catalytic activity of the MOF at the applied reaction conditions.

## 4. Conclusions

A MOF material, Cu(II)-PEIP, produced in a one-pot self-assembly process, is demonstrated for the first time to efficiently catalyze a [4 + 2]-cycloaddition between a range of azachalcone dienophiles and cyclopentadiene. This reaction is green, given that it takes place quantitatively in water in the presence of a surfactant, and nearly eliminates the use of harmful organic solvents in the workup. The MOF catalyst has shown excellent efficiency (>98% conversions) along with remarkable endo-to-exo selectivities, especially in cases of electron-deficient dienophiles bearing carboxy and trimethylammonium substituents. These results point towards an underlying role of strategically positioned Lewis-basic or Lewis-acidic moieties in the structure of the (dienophile) substrates, which enable the development of additional key interactions with the MOF catalyst, thus promoting a productive transition state. The high porosity of this MOF catalyst was essential for allowing the formation of bulky, bicyclic Diels–Alder adducts, which are reported for the first time in relation to MOF catalysis. Furthermore, the observed retention of crystallinity of the MOF by PXRD, after four reaction cycles, renders this material a potentially recyclable and precious new catalyst for Diels–Alder transformations, comparable to or even more efficient than standard homogeneous Cu(II) catalysts. Undoubtedly, this material will be useful for a number of applications in the field of D-A catalysis, where an elevated interest in material-based catalysts has been registered in recent years [42]. Finally, this MOF catalyst could be of broader scope by potentially extending its use to other organic transformations that require Lewis acid catalysis, such as addition and conjugate addition to carbonyls, carbonyl-ene reaction, various 1,3-dipolar cycloadditions leading to valuable 5-member *N*,*O*-heterocycles, and inverse electron demand D-A, among others.

## Figures and Tables

**Table 1:** Effect of various solvents/additives on MOF solubility, and conversion of the model D-A reaction between substrates **1a** and **2**. The reactions were monitored qualitatively by TLC, and quantitatively by product isolation, mass determination, and ^1^H NMR analysis. The highlighted entry (#12) indicates the reaction conditions chosen for heterogeneous catalysis on all D-A substrates. [Fixed parameters: r.t., 24 h, 2% mol Cu(II) from MOF, dienophile C = 0.25 M, diene:dienophile molar ratio = 10:1, solvent V = 1 mL].

Entry	Solvent/Additive	Solubility of Cu(II)-PEIP	Conversion (%)
1	CH_3_CN	Insoluble	8
2	CH_3_CN/H_2_O (1:10 *v*/*v*)	Insoluble	63
3	H_2_O	Insoluble	99
4	DCM	Insoluble	29
5	MeOH	Insoluble	28
6	Sat. aqueous NaCl	Insoluble	10
7	NMP	Insoluble	trace
8	BME	Insoluble	7
9	EtOH	Insoluble	10
10	DMF	Insoluble	trace
11	H_2_O/25% mol SDS	Insoluble	99
12	H_2_O/10% mol SDS	Insoluble	99
13	H_2_O/5% mol SDS	Insoluble	99

**Table 2:** Control D-A reactions in solution on the model substrates system **1a** + **2** in the absence of MOF in H_2_O/10% mol SDS at r.t. for 24 h, assessed in order to detect any ability of the individual MOF components (Entries 1–3) to serve as catalysts. The additive-free, uncatalyzed reaction (substrates only, Entry 4) was also included.

Entry	MOF Component	Conversion (%)	Endo:Exo
1	Cu(NO_3_)_2_·2.5H_2_O	35	1:1
2	PEIPH_2_	25	1:1
3	5-NH_2_-*m*BDCH_2_	29	1:1
4	None	trace	N/D

## Data Availability

Not applicable.

## Supplementary Materials

The following supporting information can be downloaded at: https://www.mdpi.com/article/10.3390/ma16155298/s1, Synthetic procedures and characterization data for all dienophiles employed in this study; Scheme S1: Aldol/dehydration reaction for azachalcones (**1a–m**) formation; Scheme S2: [4+2] (Diels-Alder) cycloadditions between azachalcones (**1a–m**) and cyclopentadiene (**2**), catalyzed by Cu(II)-PEIP MOF (heterogeneous) or Cu(OTf)_2_ (homogeneous, control); Table S1: Conditions employed in the aldol/dehydration reaction, to form azachalcones **1a–m**; Figure S1: (**a**) Paddle-wheel structure of the Cu(II) coordination sites in the MOF, showing short Cu-O (RCO_2_) bonds and long Cu-O (DMF) bonds. (**b**) Distance of a free NH_2_ from a Cu(II) catalytic site is comparable to the dimensions of the dienophile; Figure S2: ^1^H NMR of Cu(II)-PEIP, digested in HCl/DMSO: (**a**) As synthesized, prior to acetone wash. (**b**) After extensive wash with acetone and drying; Figure S3: Powder X-ray diffraction patterns of Cu(II)-PEIP catalyst, after exposure to optimized Diels-Alder reaction conditions (H_2_O/10% mol SDS), in the absence of substrates, for various times; Figure S4: ^1^H NMR spectra of the supernatant liquid, after submission of the Cu(II)-PEIP catalyst to optimized Diels-Alder reaction conditions (D_2_O/10% mol SDS), in the absence of substrates, for various times (24 h, 48 h, 72 h); Figure S5: Comparison of ^1^H NMR spectra of different batches of product **3a**, obtained from dienophile **1a** and diene **2** via Cu(II)-PEIP-catalyzed Diels-Alder reaction. (**a**–**d**) The Cu(II)-PEIP MOF catalyst was exposed to the selected aqueous conditions (D_2_O/10% mol SDS), for 0 h, 24 h, 48 h and 72 h, respectively, prior to being used in the D-A reaction.
