# Supplementary material for: Catalysis of a Diels–Alder Reaction between Azachalcones and Cyclopentadiene by a Recyclable Copper(II)-PEIP Metal-Organic Framework
Source: Materials (Basel). 2023 Jul 27;16(15):5298. doi: 10.3390/ma16155298 (PMC10419979; doi:10.3390/ma16155298)
Supplement: Supplementary file 1 [file materials-16-05298-s001.zip › materials-2503324-supplementary.pdf]

Supporting Information

Catalysis of a Diels–Alder Reaction between Azachalcones and Cyclopentadiene by a Recyclable Copper(II)-PEIP Metal-Organic Framework

Eleni Hadjikyprianou, Sotiris Petrides, Andreas Kourtellaris, Anastasios J. Tasiopoulos and Savvas N. Georgiades *

Department of Chemistry, University of Cyprus, 1 Panepistimiou Avenue, Aglandjia, 2109 Nicosia, Cyprus
* Correspondence: georgiades.savvas@ucy.ac.cy

Contents

1. Synthesis and characterization of organic compounds 2

1.1. Synthesis of dienophiles: (E)-3-(Hetero)aryl-1-(pyridin-2-yl)prop-2-en-1-ones 2

1.2. Synthesis of Diels-Alder cycloadducts: (3-(Hetero)arylbicyclo[2.2.1]hept-5-en-2-yl)-(pyridin-2-yl)methanones 11

2. Selected bond lengths and distances in the Cu(II)-PEIP MOF 19

3. Digestion of Cu(II)-PEIP MOF and ^{1}H NMR determination of residual DMF 19

4. Check of MOF stability under optimized D-A reaction conditions via PXRD 20

5. ^{1}H NMR check of aqueous supernatant for ligand leaching from MOF 20

6. Comparison of ^{1}H NMR spectra between different D-A product batches, obtained using MOF catalyst that was exposed to aqueous conditions for variable time 21

7. References 21

1. Synthesis and characterization of organic compounds

1.1. Synthesis of dienophiles: (E)-3-(Hetero)aryl-1-(pyridin-2-yl)prop-2-en-1-ones

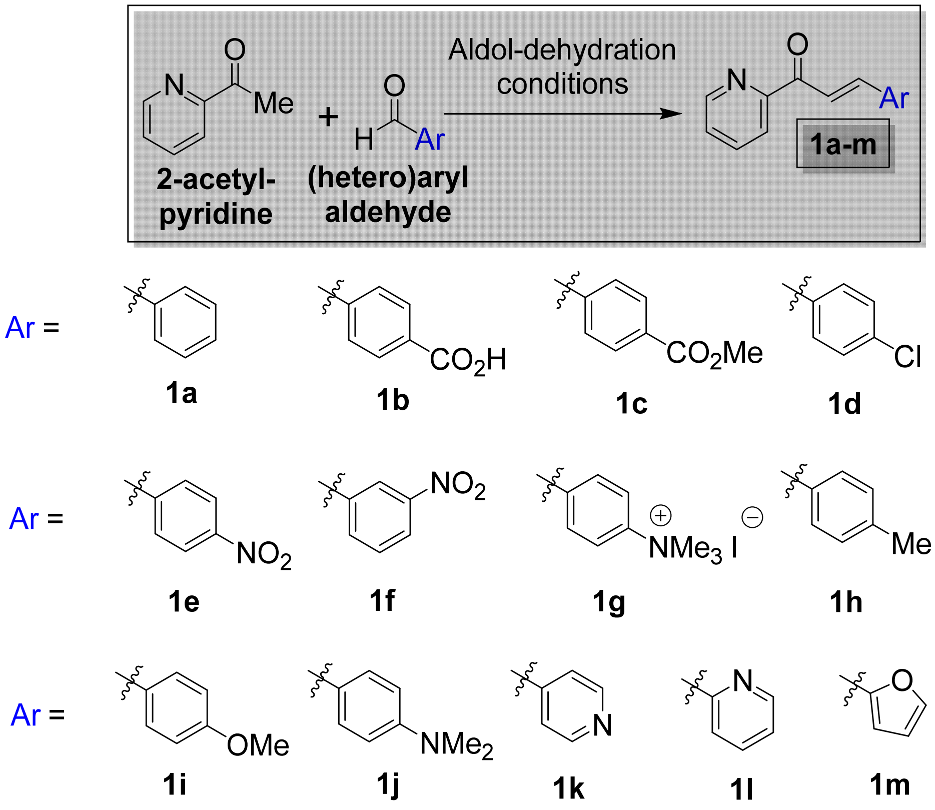

Scheme S1. Aldol/dehydration reaction for azachalcones (**1a-m**) formation.

Table S1. Conditions employed in the aldol/dehydration reaction, to form azachalcones **1a-m**. Molar ratio of 2-acetylpyridine to aldehyde was 1:1.

Entry/ prod.	Ar	Base	Base equiv.	Solvent	S.M. C (M)	Temp./ time	Yield	Ref.
**1a**	C_6H_5	NaOH	2.8	H_2O	0.09	0 then 4 °C/ 24 h	97	S1
**1b**	4-CO_2H-C_6H_5	NaOH	1.5	EtOH-H_2O (2:1)	0.54	0 °C / 2 h	89	S2

Table S1 (continued). Conditions employed in the aldol/dehydration reaction, to form azachalcones **1a-m**. Molar ratio of 2-acetylpyridine to aldehyde was 1:1.

**Entry/ prod.**	**Ar**	**Base**	**Base equiv.**	**Solvent**	**S.M. C (M)**	**Temp./ time**	**Yield**	**Ref.**
**1c**	4-CO_2Me-C_6H_5	NaOH	1.5	EtOH-H_2O (2:1)	0.54	0 °C / 2 h	50	S2
**1d**	4-Cl-C_6H_5	KOH	1.0	MeOH-H_2O (5:1)	0.41	0 °C / 2 h	94	S3
**1e**	4-NO_2-C_6H_5	KOH	1.0	MeOH-H_2O (5:1)	0.41	0 °C / 3 h	60	S3
**1f**	3-NO_2-C_6H_5	KOH	1.0	MeOH-H_2O (5:1)	0.41	0 °C / 3 h	85	S3
**1g**	4-NMe_3I-C_6H_5	KOH	1.0	MeOH	0.41	0 °C / 3 h	68	-
**1h**	4-Me-C_6H_5	NaOH	2.8	H_2O	0.09	0 then 4 °C / 24 h	99	S1
**1i**	4-OMe-C_6H_5	NaOH	2.8	H_2O	0.09	0 then 4 °C / 24 h	95	S1
**1j**	4-NMe_2-C_6H_5	KOH	5.45	EtOH-H_2O (5:3)	0.25	r.t. / 3 h	76	-
**1k**	4-pyridyl	Na_2CO_3	0.25	H_2O	0.05	45 °C / 2 h	50	S4
**1l**	2-pyridyl	Na_2CO_3	0.25	H_2O	0.05	70 °C / 2 h then 4 °C / 24 h	84	S4
**1m**	2-furyl	NaOH	1.0	MeOH-H_2O (1:1)	0.26	0 °C / 3 h	60	-

• (*E*)-3-Phenyl-1-(pyridin-2-yl)prop-2-en-1-one (**1a**)

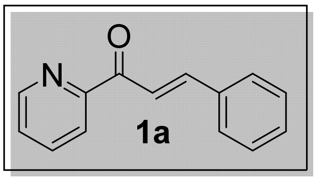

For this reaction, a variation of a literature method [S1] was employed. In a round-bottom flask containing 18 mL of H_2O, cooled at 0 °C, were introduced 2-acetylpyridine (0.20 mL, 1.8 mmol, 1.0 equiv.) and benzaldehyde (0.18 mL, 1.8 mmol, 1.0 equiv.) and the mixture was shaken thoroughly. 2 mL of 10% wt. NaOH aqueous solution (5 mmol, 2.8 equiv.) were added and the mixture was again shaken and left overnight, undisturbed at 4 °C. Subsequently, the mixture was filtered under vacuum and the obtained crude solid was washed with cold water and cold EtOH. It was further recrystallized from EtOH to afford compound **1a** (0.365 g, 1.75 mmol, 97%) as a yellow crystalline solid. Compound **1a** was characterized by ^{1}H NMR and ^{13}C NMR, and found to be free of organic impurities.

^{1}H NMR ($CDCl_3$): δ_H (ppm) 8.75 (ddd, J_1 = 4.8 Hz, J_2 = 1.8 Hz, J_3 = 0.8 Hz, 1H), 8.31 (d, J = 16.1 Hz, 1H), 8.20 (dd, J_1 = 7.8 Hz, J_2 = 1.1 Hz, 1H), 7.95 (d, J = 16.1 Hz, 1H), 7.89 (app. dt, J_1 = 7.7 Hz, J_2 = 1.8 Hz, 1H), 7.74 (dd, J_1 = 6.5 Hz, J_2 = 2.0 Hz, 2H), 7.50 (ddd, J_1 = 7.6 Hz, J_2 = 4.8 Hz, J_3 = 1.1 Hz, 1H), 7.43-7.41 (m, 2 signals overlapping, 3H). ^{13}C NMR ($CDCl_3$): δ_C (ppm) 189.7, 154.4, 149.0, 145.0, 137.2, 135.3, 130.7, 129.0, 128.9, 127.0, 123.1, 121.0. MS (ES-API), m/z: calcd for $C_{14}H_{11}NO$: 209.08; found: 210.0 [$M+H^+$], 231.9 [$M+Na^+$].

• (*E*)-4-(3-Oxo-3-(pyridin-2-yl)prop-1-en-1-yl)benzoic acid (**1b**)

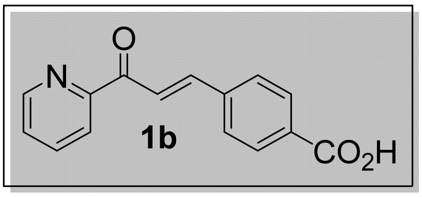

For this reaction, a variation of a literature method [S2] was employed. In a round-bottom flask containing 2.6 mL of EtOH, cooled at 0 °C, were introduced 4-formylbenzoic acid (0.315 g, 2.1 mmol, 1.0 equiv.), 1.3 mL of a 10% NaOH aqueous solution (3.25 mmol, 1.5 equiv.) and 2-acetylpyridine (0.23 mL, 2.1 mmol, 1.0 equiv.). The mixture was vigorously stirred at 0 °C for 2 h and was subsequently filtered under vacuum. The collected crude solid was dissolved in a small amount of water and the solution was acidified with 1 M aqueous HCl, to pH 2. The mixture was filtered under vacuum and the collected solid was washed with cold EtOH, to afford compound **1b** (0.474 g, 1.87 mmol, 89%) as a white powder. Compound **1b** was characterized by ^{1}H NMR and ^{13}C NMR, and found to be free of organic impurities.

^{1}H NMR (DMSO): δ_H (ppm) 8.82 (dd, J_1 = 4.7 Hz, J_2 = 1.7 Hz, 1H), 8.35 (d, J = 16.1 Hz, 1H), 8.15 (dd, J_1 = 7.6 Hz, J_2 = 1.2 Hz, 1H), 8.08 (app. dt, J_1 = 7.5 Hz, J_2 = 1.7 Hz, 1H), 8.01

(d, J = 8.3 Hz, 2H), 7.93 (d, J = 8.3 Hz, 2H), 7.89 (d, J = 16.1 Hz, 1H), 7.73 (ddd, J_1 = 7.4 Hz, J_2 = 4.7 Hz, J_3 = 1.2 Hz, 1H). ^{13}C NMR (DMSO): δ_C (ppm) 188.7, 169.0, 153.5, 149.2, 144.2, 143.0, 137.8, 134.6, 129.7, 127.9, 127.7, 122.5, 120.5. MS (ES-API), m/z: calcd for $C_{15}H_{11}NO_3$: 253.07; found: 254.0 [M+H$^+$], 251.9 [M-H$^+$].

• Methyl (*E*)-4-(3-Oxo-3-(pyridin-2-yl)prop-1-en-1-yl)benzoate (**1c**)

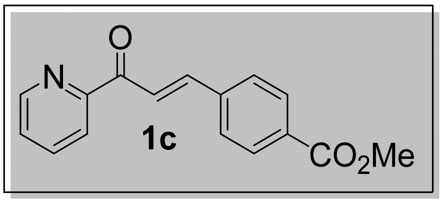

For this reaction, a variation of a literature method [S2] was employed. In a round-bottom flask containing 2.6 mL of EtOH, cooled at 0 °C, were introduced methyl 4-formylbenzoate (0.345 g, 2.1 mmol, 1.0 equiv.), 1.3 mL of a 10% NaOH aqueous solution (3.25 mmol, 1.5 equiv.) and 2-acetylpyridine (0.23 mL, 2.1 mmol, 1.0 equiv.). The mixture was vigorously stirred at 0 °C for 2 h, and was subsequently filtered under vacuum. The collected crude solid was washed with cold EtOH, to afford compound **1c** (0.281 g, 1.05 mmol, 50%) as a yellow powder. Compound **1c** was characterized by ^{1}H NMR and ^{13}C NMR, and found to be free of organic impurities.

^{1}H NMR (CDCl$_3$): δ_H (ppm) 8.76 (ddd, J_1 = 4.8 Hz, J_2 = 1.8 Hz, J_3 = 0.9 Hz, 1H), 8.38 (d, J = 16.1 Hz, 1H), 8.20 (dd, J_1 = 7.9 Hz, J_2 = 1.1 Hz, 1H), 8.08 (d, J = 8.4 Hz, 2H), 7.93 (d, J = 16.1 Hz, 1H), 7.90 (app. dt, J_1 = 7.8 Hz, J_2 = 1.8 Hz, 1H), 7.79 (d, J = 8.4 Hz, 2H), 7.51 (ddd, J_1 = 7.7 Hz, J_2 = 4.8 Hz, J_3 = 1.1 Hz, 1H) 3.94 (s, 3H). ^{13}C NMR (CDCl$_3$): δ_C (ppm) 189.4, 166.7, 154.1, 149.1, 143.2, 139.5, 137.3, 131.6, 130.2, 128.7, 127.2, 123.2, 123.1, 52.4. MS (ES-API), m/z: calcd for $C_{16}H_{13}NO_3$: 267.09; found: 268.0 [M+H$^+$], 290.0 [M+Na$^+$].

• (*E*)-3-(4-Chlorophenyl)-1-(pyridin-2-yl)prop-2-en-1-one (**1d**)

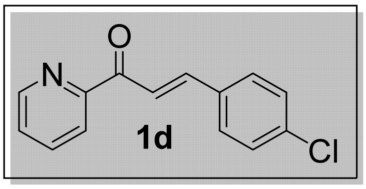

For this reaction, a variation of a literature method [S3] was employed. In a round-bottom flask containing 5.1 mL of a 5:1 MeOH-H_2O mixture, cooled at 0 °C, were introduced 4-chlorobenzaldehyde (0.295 g, 2.1 mmol, 1.0 equiv.), KOH (0.118 g, 2.1 mmol, 1.0 equiv.) and 2-acetylpyridine (0.23 mL, 2.1 mmol, 1.0 equiv.). The mixture was vigorously stirred at 0 °C for 2 h and was subsequently filtered under vacuum. The crude solid was thoroughly washed with cold EtOH, to

afford compound **1d** (0.48 g, 1.97 mmol, 94%) as a grey powder. Compound **1d** was characterized by ^{1}H NMR and ^{13}C NMR, and found to be free of organic impurities.

^{1}H NMR ($CDCl_3$): δ_H (ppm) 8.74 (ddd, J_1 = 4.8 Hz, J_2 = 1.8 Hz, J_3 = 0.8 Hz, 1H), 8.28 (d, J = 16.1 Hz, 1H), 8.19 (dd, J_1 = 7.9 Hz, J_2 = 1.1 Hz, 1H), 7.88 (app. dt, J_1 = 7.8 Hz, J_2 = 1.8 Hz, 1H), 7.87 (d, J = 16.1 Hz, 1H), 7.66 (d, J = 8.5 Hz, 2H), 7.50 (ddd, J_1 = 7.7 Hz, J_2 = 4.8 Hz, J_3 = 1.1 Hz, 1H), 7.39 (d, J = 8.5 Hz, 2H). ^{13}C NMR ($CDCl_3$): δ_C (ppm) 189.4, 154.2, 149.0, 143.3, 137.2, 136.6, 133.8, 130.1, 129.3, 127.2, 123.1, 121.5. MS (ES-API), m/z: calcd for $C_{14}H_{10}ClNO$: 243.05; found: 243.9 [M+H$^+$].

• (*E*)-3-(4-Nitrophenyl)-1-(pyridin-2-yl)prop-2-en-1-one (**1e**)

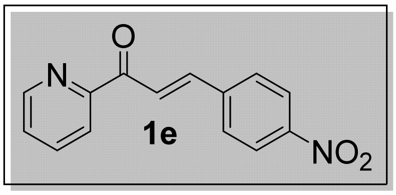

For this reaction, a literature method [S3] was employed. In a round-bottom flask containing 5.1 mL of a 5:1 MeOH-H_2O mixture, cooled at 0 °C, were introduced KOH (0.118 g, 2.1 mmol, 1.0 equiv.), 4-nitrobenzaldehyde (0.317 g, 2.1 mmol, 1.0 equiv.) and 2-acetylpyridine (0.23 mL, 2.1 mmol, 1.0 equiv.). The mixture was vigorously stirred at 0 °C for 3 h and was subsequently filtered under vacuum. The crude solid was thoroughly washed with cold MeOH and then recrystallized from EtOH, to afford compound **1e** (0.32 g, 1.26 mmol, 60%) as a beige powder. Compound **1e** was characterized by ^{1}H NMR and ^{13}C NMR, and found to be free of organic impurities.

^{1}H NMR ($CDCl_3$): δ_H (ppm) 8.76 (d, J = 3.3 Hz, 1H), 8.43 (d, J = 16.2 Hz, 1H), 8.27 (d, J = 8.4 Hz, 2H), 8.21 (d, J = 7.8 Hz, 1H), 7.97-7.89 (m, 2 signals overlapping, 2H), 7.87 (d, J = 8.4 Hz, 2H), 7.54 (m, 1H). ^{13}C NMR ($CDCl_3$): δ_C (ppm) 189.1, 153.7, 149.1, 148.7, 141.5, 141.4, 137.4, 129.4, 127.5, 124.9, 124.3, 123.2. MS (ES-API), m/z: calcd for $C_{14}H_{10}N_2O_3$: 254.07; found: 255.0 [M+H$^+$].

• (*E*)-3-(3-Nitrophenyl)-1-(pyridin-2-yl)prop-2-en-1-one (**1f**)

For this reaction, a literature method [S3] was employed. In a round-bottom flask containing 5.1 mL of a 5:1 MeOH-H_2O mixture, cooled at 0 °C, were introduced KOH (0.118 g, 2.1 mmol, 1.0 equiv.), 3-nitrobenzaldehyde (0.317 g, 2.1 mmol, 1.0 equiv.) and 2-acetylpyridine (0.23 mL, 2.1 mmol, 1.0 equiv.). The mixture was vigorously stirred at 0 °C for 3 h and was subsequently filtered under vacuum. The crude solid was

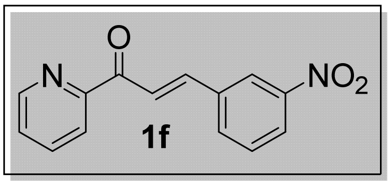

thoroughly washed with cold MeOH, to afford compound **1f** (0.453 g, 1.785 mmol, 85%) as a brown powder. Compound **1f** was characterized by ^{1}H NMR and ^{13}C NMR, and found to be free of organic impurities.

^{1}H NMR ($CDCl_3$): δ_H (ppm) 8.77 (d, J = 3.4 Hz, 1H), 8.58 (s, 1H), 8.43 (d, J = 16.1 Hz, 1H), 8.25 (d, J = 8.1 Hz, 1H), 8.20 (d, J = 8.1 Hz, 1H), 8.00 (d, J = 7.6 Hz, 1H), 7.93 (d, J = 16.1 Hz, 1H), 7.91 (m, 1H), 7.61 (app. t, J = 8.1 Hz, 1H), 7,53 (app. t, J = 5.4 Hz, 1H). ^{13}C NMR ($CDCl_3$): δ_C (ppm) 189.2, 153.8, 149.1, 148.9, 141.6, 137.3, 137.1, 134.6, 130.1, 127.5, 124.8, 123.8, 123.2, 123.0. MS (ES-API), m/z: calcd for $C_{14}H_{10}N_2O_3$: 254.07; found: 255.0 [M+H$^+$].

• (*E*)-*N*,*N*,*N*-Trimethyl-4-(3-oxo-3-(pyridin-2-yl)prop-1-en-1-yl)benzenaminium iodide (**1g**)

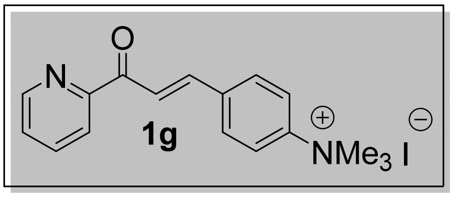

In a round-bottom flask containing 5.1 mL of MeOH, cooled at 0 °C, were introduced KOH (0.118 g, 2.1 mmol, 1.0 equiv.), 4-formyl-*N*,*N*,*N*-trimethylbenzenaminium iodide (0,611 g, 2.1 mmol, 1.0 equiv.) and 2-acetylpyridine (0.23 mL, 2.1 mmol, 1.0 equiv.). The mixture was vigorously stirred at 0 °C for 3 h and was subsequently filtered under vacuum. The crude solid was thoroughly washed with cold MeOH, then recrystallized from EtOH, to afford compound **1g** (0,564 g, 1.43 mmol, 68%) as a bright yellow powder. Compound **1g** was characterized by ^{1}H NMR and ^{13}C NMR, and found to be free of organic impurities.

^{1}H NMR (D_2O): δ_H (ppm) 8.68 (ddd, J_1 = 4.8 Hz, J_2 = 1.8 Hz, J_3 = 1.0 Hz, 1H), 8.10 (dd, J_1 = 7.8 Hz, J_2 = 1.2 Hz, 1H), 8.05 (app. dt, J_1 = 7.7 Hz, J_2 = 1.8 Hz, 1H), 7.99 (d, J = 16.1 Hz, 1H), 7.94 (d, J = 9.1 Hz, 2H), 7.89 (d, J = 9.1 Hz, 2H), 7.83 (d, J = 16.1 Hz, 1H), 7.68 (ddd, J_1 = 7.6 Hz, J_2 = 4.8 Hz, J_3 = 1.2 Hz, 1H), 3.66 (s, 9H). ^{13}C NMR (D_2O): δ_C (ppm) 192.1, 152.7, 148.9, 147.7, 143.8, 138.6, 136.4, 130.4, 128.2, 124.0, 123.8, 120.4, 56.9. MS (ES-API), m/z: calcd for $C_{17}H_{19}N_2O^+$: 267.15; found: 267.0 [M].

• (*E*)-1-(Pyridin-2-yl)-3-(*p*-tolyl)prop-2-en-1-one (**1h**)

For this reaction, a variation of a literature method [S1] was employed. In a round-bottom flask containing 18 mL of H_2O, cooled at 0 °C, were introduced 2-acetylpyridine (0.20 mL, 1.8 mmol, 1.0 equiv.) and 4-methylbenzaldehyde (0.21 mL,

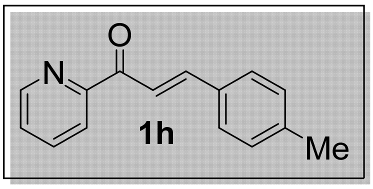

1.8 mmol, 1.0 equiv.) and the mixture was shaken thoroughly. 2 mL of 10% wt. NaOH aqueous solution (5 mmol, 2.8 equiv.) were added and the mixture was again shaken and left overnight, undisturbed at 4 °C. Subsequently, the mixture was filtered under vacuum and the obtained crude solid was washed with cold water and cold EtOH. It was further recrystallized from EtOH to afford compound **1h** (0.40 g, 1.79 mmol, 99%) as a yellow crystalline solid. Compound **1h** was characterized by ^{1}H NMR and ^{13}C NMR, and found to be free of organic impurities.

^{1}H NMR (CDCl$_3$): δ_H (ppm) 8.74 (d, *J* = 4.9 Hz, 1H), 8.26 (d, *J* = 16.1 Hz, 1H), 8.18 (d, *J* = 7.9 Hz, 1H), 7.93 (d, *J* = 16.1 Hz, 1H), 7.86 (app. t, *J* = 7.8 Hz, 1H), 7.63 (d, *J* = 7.8 Hz, 2H), 7.48 (ddd, J_1 = 7.7 Hz, J_2 = 4.9 Hz, J_3 = 1.1 Hz, 1H), 7.22 (d, *J* = 7.8 Hz, 2H), 2.38 (s, 3H). ^{13}C NMR (CDCl$_3$): δ_C (ppm) 189.6, 154.5, 148.9, 145.1, 141.2, 137.1, 132.5, 129.7, 129.0, 126.9, 123.0, 119.9, 21.7. MS (ES-API), m/z: calcd for $C_{15}H_{13}NO$: 223.10; found: 223.9 [M+H$^+$].

• (*E*)-3-(4-Methoxyphenyl)-1-(pyridin-2-yl)prop-2-en-1-one (**1i**)

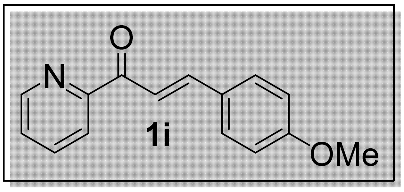

For this reaction, a variation of a literature method [S1] was employed. In a round-bottom flask containing 18 mL of H_2O, cooled at 0 °C, were introduced 2-acetylpyridine (0.20 mL, 1.8 mmol, 1.0 equiv.) and 4-methoxylbenzaldehyde (0.22 mL, 1.8 mmol, 1.0 equiv.) and the mixture was shaken thoroughly. 2 mL of 10% wt. NaOH aqueous solution (5 mmol, 2.8 equiv.) were added and the mixture was again shaken and left overnight, undisturbed at 4 °C. Subsequently, the mixture was filtered under vacuum and the obtained crude solid was washed with cold water and cold EtOH. It was further recrystallized from EtOH to afford compound **1i** (0.409 g, 1.71 mmol, 95%) as a yellow crystalline solid. Compound **1i** was characterized by ^{1}H NMR and ^{13}C NMR, and found to be free of organic impurities.

^{1}H NMR (CDCl$_3$): δ_H (ppm) 8.76 (ddd, J_1 = 4.8 Hz, J_2 = 1.8 Hz, J_3 = 0.8 Hz, 1H), 8.22 (dd, J_1 = 7.8 Hz, J_2 = 1.1 Hz, 1H), 8.21 (d, *J* = 16.0 Hz, 1H), 7.94 (d, *J* = 16.0 Hz, 1H), 7.91 (app. dt, J_1 = 7.7 Hz, J_2 = 1.8 Hz, 1H), 7.72 (d, *J* = 8.7 Hz, 2H), 7.52 (ddd, J_1 = 7.6 Hz, J_2 = 4.8 Hz, J_3 = 1.1 Hz, 1H), 6.94 (d, *J* = 8.7 Hz, 2H), 3.86 (s, 3H). ^{13}C NMR (CDCl$_3$): δ_C (ppm) 189.5, 161.9, 154.6, 148.9, 144.9, 137.2, 130.8, 128.1, 126.9, 123.0, 118.6, 114.4, 55.5. MS (ES-API), m/z: calcd for $C_{15}H_{13}NO_2$: 239.09; found: 240.0 [M+H$^+$], 262.0 [M+Na$^+$].

• (*E*)-3-(4-(Dimethylamino)phenyl)-1-(pyridin-2-yl)prop-2-en-1-one (**1j**)

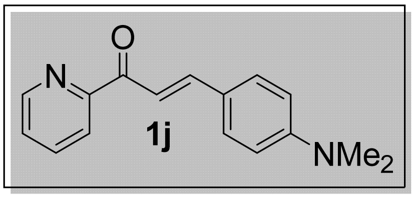

In a round-bottom flask containing 5.55 mL of EtOH, at r.t., were introduced 2-acetylpyridine (0.23 mL, 2.2 mmol, 1.0 equiv.) and 4-(dimethylamino) benzaldehyde (0.328 g, 2.2 mmol, 1.0 equiv.), and the mixture was shaken thoroughly. 3.33 mL of 20% wt. KOH aqueous solution (12 mmol, 5.45 equiv.) were then added and the mixture was stirred at r.t. for 3 h. Finally, 10 mL of H_2O were added to the mixture, followed by filtration under vacuum. The obtained crude solid was washed with cold water and cold EtOH. It was further recrystallized from DCM-hexane, to afford compound **1j** (0.421 g, 1.67 mmol, 76%) as a bright orange crystalline solid. Compound **1j** was characterized by ^{1}H NMR and ^{13}C NMR, and found to be free of organic impurities.

^{1}H NMR ($CDCl_3$): δ_H (ppm) 8.73 (ddd, J_1 = 4.8 Hz, J_2 = 1.8 Hz, J_3 = 0.9 Hz, 1H), 8.18 (dd, J_1 = 7.8 Hz, J_2 = 1.1 Hz, 1H), 8.08 (d, J = 15.9 Hz, 1H), 7.93 (d, J = 15.9 Hz, 1H), 7.85 (app. dt, J_1 = 7.7 Hz, J_2 = 1.8 Hz, 1H), 7.64 (d, J = 8.9 Hz, 2H), 7.45 (ddd, J_1 = 7.6 Hz, J_2 = 4.8 Hz, J_3 = 1.1 Hz, 1H), 6.69 (d, J = 8.9 Hz, 2H), 3.04 (s, 6H). ^{13}C NMR ($CDCl_3$): δ_C (ppm) 189.4, 155.1, 152.2, 148.8, 146.2, 137.0, 131.1, 126.5, 123.2, 122.9, 115.6, 111.9, 40.3. MS (ES-API), m/z: calcd for $C_{16}H_{16}N_2O$: 252.13; found: 253.0 [M+H$^+$], 275.0 [M+Na$^+$].

• (*E*)-1-(Pyridin-2-yl)-3-(pyridin-4-yl)prop-2-en-1-one (**1k**)

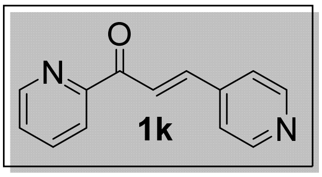

For this reaction, a variation of a literature method [S4] was employed. In a round-bottom flask containing 35 mL of H_2O, were introduced 4-pyridinecarboxaldehyde (0,236 g, 2.2 mmol, 1.0 equiv.) and 2-acetylpyridine (0.24 mL, 2.2 mmol, 1.0 equiv.). The mixture was briefly stirred in a pre-heated oil bath at 45 °C, followed by addition of a Na_2CO_3 (0.058 g, 0,55 mmol, 0.25 equiv.) solution in 10 mL of H_2O. Stirring continued at 45 °C for 2 h, leading to formation of an off-yellow precipitate. The mixture was filtered under vacuum and the crude solid was washed with cold H_2O. It was redissolved in DCM and applied to a silica column for flash chromatography. Isocratic elution took place, using a hexane-EtOAc 1:3 mixture, to afford compound **1k** (0.231 g, 1.1 mmol, 50%) as a yellow powder. Compound **1k** was characterized by ^{1}H NMR and ^{13}C NMR, and found to be free of organic impurities.

^{1}H NMR ($CDCl_3$): δ_H (ppm) 8.75 (ddd, J_1 = 4.8 Hz, J_2 = 1.8 Hz, J_3 = 0.9 Hz, 1H), 8.68 (d, J = 6.0 Hz, 2H), 8.46 (d, J = 16.1 Hz, 1H), 8.19 (dd, J_1 = 7.8 Hz, J_2 = 1.1 Hz, 1H), 7.90 (app. dt, J_1 = 7.7 Hz, J_2 = 1.8 Hz, 1H), 7.81 (d, J = 16.1 Hz, 1H), 7.56 (d, J = 6.0 Hz, 2H), 7.52

(ddd, J_1 = 7.6 Hz, J_2 = 4.8 Hz, J_3 = 1.1 Hz, 1H). ^{13}C NMR ($CDCl_3$): δ_C (ppm) 189.2, 153.7, 150.5, 149.1, 142.7, 141.3, 137.3, 127.5, 125.4, 123.2, 122.5. MS (ES-API), m/z: calcd for $C_{13}H_{10}N_2O$: 210.08; found: 210.9 [$M+H^+$].

• (*E*)-1,3-Di(pyridin-2-yl)prop-2-en-1-one (**1l**)

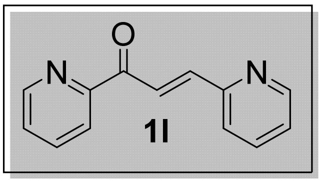

For this reaction, a variation of a literature method [S4] was employed. In a round-bottom flask containing 35 mL of H_2O, were introduced 2-pyridinecarboxaldehyde (0,21 mL, 2.2 mmol, 1.0 equiv.) and 2-acetylpyridine (0.24 mL, 2.2 mmol, 1.0 equiv.). The mixture was briefly stirred in a pre-heated oil bath at 45 °C, followed by addition of a Na_2CO_3 (0.058 g, 0,55 mmol, 0.25 equiv.) solution in 10 mL of H_2O. Stirring continued at 70 °C for 2 h. The mixture was cooled down to r.t., and left overnight at 4 °C. It was then filtered under vacuum, and the crude solid was washed with cold H_2O, to afford compound **1l** (0.389 g, 1.85 mmol, 84%) as a yellow powder. Compound **1l** was characterized by ^1H NMR and ^{13}C NMR, and found to be free of organic impurities.

^1H NMR ($CDCl_3$): δ_H (ppm) 8.76 (d, J = 4.8 Hz, 1H), 8.72 (d, J = 4.8 Hz, 1H), 8.69 (d, J = 15.9 Hz, 1H), 8.19 (d, J = 7.8 Hz, 1H), 7.92 (d, J = 15.9 Hz, 1H), 7.88 (app. dt, J_1 = 7.7 Hz, J_2 = 1.7 Hz, 1H), 7.75 (app. dt, J_1 = 7.7 Hz, J_2 = 1.5 Hz, 1H), 7.62 (d, J = 7.8 Hz, 1H), 7.50 (ddd, J_1 = 7.6 Hz, J_2 = 4.8 Hz, J_3 = 0.8 Hz, 1H), 7.30 (dd, J_1 = 7.6 Hz, J_2 = 4.8 Hz, 1H). ^{13}C NMR ($CDCl_3$): δ_C (ppm) 188.08, 154.02, 151.96, 149.57, 149.21, 141.85, 137.14, 127.22, 124.90, 124.60, 123.09, 122.06, 121.87. MS (ES-API), m/z: calcd for $C_{13}H_{10}N_2O$: 210.08; found: 210.9 [$M+H^+$].

• (*E*)-3-(Furan-2-yl)-1-(pyridin-2-yl)prop-2-en-1-one (**1m**)

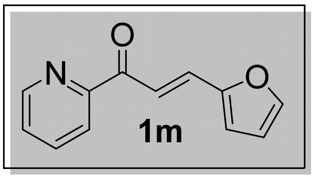

In a round-bottom flask containing 8 mL of a 1:1 MeOH-H_2O mixture, cooled at 0 °C, were introduced NaOH (0.084 g, 2.1 mmol, 1.0 equiv.), furfural (0.17 mL, 2.1 mmol, 1.0 equiv.) and 2-acetylpyridine (0.23 mL, 2.1 mmol, 1.0 equiv.). The mixture was vigorously stirred at 0 °C for 3 h and was subsequently filtered under vacuum. The crude solid was thoroughly washed with cold MeOH, then recrystallized from EtOH, to afford compound **1m** (0.251 g, 1.26 mmol, 60%) as a brown powder. Compound **1m** was characterized by ^1H NMR and ^{13}C NMR, and found to be free of organic impurities.

^{1}H NMR ($CDCl_3$): δ_H (ppm) 8.76 (d, J = 4.8 Hz, 1H), 8.18 (d, J = 7.8 Hz, 1H), 8.14 (d, J = 15.8 Hz, 1H), 7.89 (app. dt, J_1 = 7.7 Hz, J_2 = 1.7 Hz, 1H), 7.71 (d, J = 15.8 Hz, 1H), 7.55 (d, J = 1.7 Hz, 1H), 7.50 (ddd, J_1 = 7.6 Hz, J_2 = 4.8 Hz, J_3 = 1.1 Hz, 1H), 6.79 (d, J = 3.5 Hz, 1H), 6.52 (dd, J_1 = 3.5 Hz, J_2 = 1.7 Hz, 1H). ^{13}C NMR ($CDCl_3$): δ_C (ppm) 189.3, 154.2, 152.1, 148.9, 145.2, 137.0, 130.7, 126.8, 122.8, 118.7, 116.3, 112.7. MS (ES-API), m/z: calcd for $C_{12}H_9NO_2$: 199.06; found: 199.9 [M+H$^+$], 221.9 [M+Na$^+$].

1.2. Synthesis of Diels-Alder cycloadducts: (3-(Hetero)arylbicyclo[2.2.1]hept-5-en-2-yl)(pyridin-2-yl)methanones

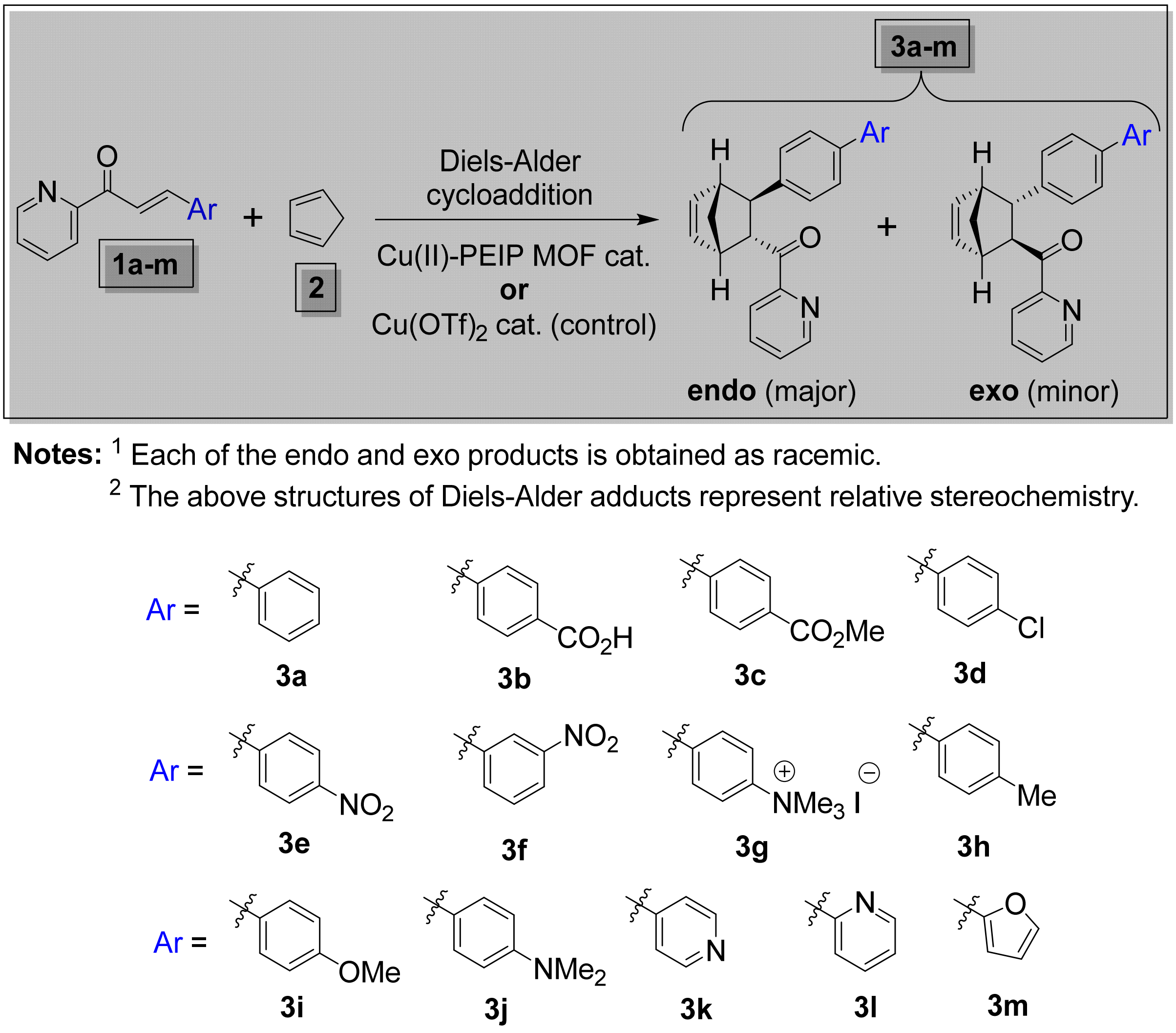

Scheme S2. [4+2] (Diels-Alder) cycloadditions between azachalcones (**1a-m**) and cyclopentadiene (**2**), catalyzed by Cu(II)-PEIP MOF (heterogeneous) or Cu(OTf)$_2$ (homogeneous, control).

• (3-Phenylbicyclo[2.2.1]hept-5-en-2-yl)(pyridin-2-yl)methanone (**3a**)

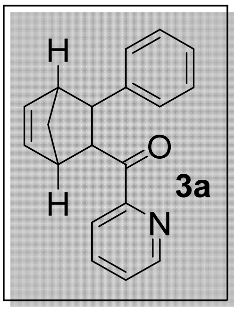

Product **3a** was prepared according to the general methods for the D-A cycloaddition described in the manuscript. Cu(II)-PEIP MOF-catalyzed reaction: 99% conversion, 8:1 mol. ratio of endo:exo. $Cu(OTf)_2$-catalyzed reaction: 99% conversion, 9:1 mol. ratio of endo:exo. Only the endo product was characterized.

Endo: ^{1}H NMR ($CDCl_3$): δ_H (ppm) 8.72 (ddd, J_1 = 4.7 Hz, J_2 = 1.8 Hz, J_3 = 0.9 Hz, 1H), 8.06 (dd, J_1 = 7.9 Hz, J_2 = 1.1 Hz, 1H), 7.86 (app. dt, J_1 = 7.8 Hz, J_2 = 1.8 Hz, 1H), 7.49 (ddd, J_1 = 7.7 Hz, J_2 = 4.7 Hz, J_3 = 1.1 Hz, 1H), 7.37 (d, J = 7.9 Hz, 2H), 7.32 (app. t, J = 7.6 Hz, 2H), 7.21 (tt, J_1 = 7.3 Hz, J_2= 1.3 Hz, 1H), 6.55 (dd, J_1 = 5.6 Hz, J_2 = 3.2 Hz, 1H), 5.88 (dd, J_1 = 5.6 Hz, J_2 = 2.8 Hz, 1H), 4.58 (dd, J_1 = 5.1 Hz, J_2 = 3.6 Hz, 1H), 3.60 (bs, 1H), 3.51 (d, J = 5.1 Hz, 1H), 3.14 (dt, J_1 = 3.4 Hz, J_2 = 1.7 Hz, 1H), 2.12 (d, J = 8.5 Hz, 1H), 1.66 (ddd, J_1 = 8.5 Hz, J_2 = 3.6 Hz, J_3 = 1.7 Hz, 1H). ^{13}C NMR ($CDCl_3$): δ_C (ppm) 201.2, 153.7, 149.0, 144.7, 139.5, 136.9, 133.0, 128.5, 127.8, 127.0, 125.9, 122.3, 54.4, 49.5, 48.9, 48.3, 45.7. MS (ES-API), m/z: calcd for $C_{19}H_{17}NO$: 275.13; found: 276.0 [$M+H^+$], 298.0 [$M+Na^+$].

• 4-(3-Picolinoylbicyclo[2.2.1]hept-5-en-2-yl)benzoic acid (**3b**)

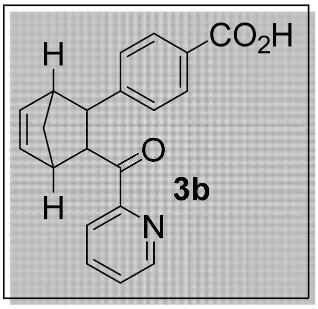

Product **3b** was prepared according to the general methods for the D-A cycloaddition described in the manuscript. Cu(II)-PEIP MOF-catalyzed reaction: 99% conversion, endo only. $Cu(OTf)_2$-catalyzed reaction: 99% conversion, 9:1 mol. ratio of endo:exo. Only the endo product was characterized.

Endo: ^{1}H NMR ($CDCl_3$): δ_H (ppm) 8.68 (ddd, J_1 = 4.7 Hz, J_2 = 1.8 Hz, J_3 = 0.9 Hz, 1H), 8.02 (dd, J_1 = 7.8 Hz, J_2 = 1.2 Hz, 1H), 7.99 (d, J = 8.3 Hz, 2H), 7.83 (app. dt, J_1 = 7.7 Hz, J_2 = 1.8 Hz, 1H), 7.47 (ddd, J_1 = 7.6 Hz, J_2 = 4.7 Hz, J_3= 1.2 Hz, 1H), 7.40 (d, J = 8.3 Hz, 2H), 6.49 (dd, J_1 = 5.6 Hz, J_2 = 3.2 Hz, 1H), 5.85 (dd, J_1 = 5.6 Hz, J_2 = 2.8 Hz, 1H), 4.51 (dd, J_1 = 5.2 Hz, J_2 = 3.6 Hz, 1H), 3.57 (bs, 1H), 3.51 (d, J = 5.2 Hz, 1H), 3.12 (dt, J_1 = 3.4 Hz, J_2 = 1.7 Hz, 1H), 2.06 (d, J = 8.5 Hz, 1H), 1.64 (ddd, J_1 = 8.5 Hz, J_2 = 3.6 Hz, J_3 = 1.7 Hz, 1H). ^{13}C NMR ($CDCl_3$): δ_C (ppm) 200.7, 171.4, 153.4, 151.2, 148.9, 139.2, 137.1, 133.2, 130.4, 127.8, 127.1, 127.0, 122.4, 54.5, 49.1, 48.8, 48.2, 45.8. MS (ES-API), m/z: calcd for $C_{20}H_{17}NO_3$: 319.12; found: 320.0 [$M+H^+$], 342.0 [$M+Na^+$], 318.0 [$M-H^+$].

• Methyl 4-(3-picolinoylbicyclo[2.2.1]hept-5-en-2-yl)benzoate (**3c**)

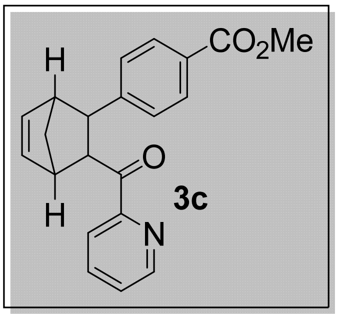

Product **3c** was prepared according to the general methods for the D-A cycloaddition described in the manuscript. Cu(II)-PEIP MOF-catalyzed reaction: 99% conversion, 7:1 mol. ratio of endo:exo. $Cu(OTf)_2$-catalyzed reaction: 99% conversion, 6:1 mol. ratio of endo:exo. Only the endo product was characterized.

Endo: ^{1}H NMR ($CDCl_3$): δ_H (ppm) 8.66 (d, J = 4.3 Hz, 1H), 8.01 (d, J = 7.9 Hz, 1H), 7.94 (d, J = 8.3 Hz, 2H), 7.82 (dt, J_1 = 7.7 Hz, J_2 = 1.6 Hz, 1H), 7.45 (ddd, J_1 = 7.8 Hz, J_2 = 4.8 Hz, J_3 = 0.7 Hz, 1H), 7.37 (d, J = 8.3 Hz, 2H), 6.48 (dd, J_1 = 5.6 Hz, J_2 = 3.2 Hz, 1H), 5.84 (dd, J_1 = 5.6 Hz, J_2 = 2.8 Hz, 1H), 4.50 (dd, J_1 = 5.3 Hz, J_2 = 3.6 Hz, 1H), 3.88 (s, 3H), 3.57 (bs, 1H), 3.49 (d, J = 5.3 Hz, 1H), 3.11 (dt, J_1 = 3.4 Hz, J_2 = 1.8 Hz, 1H), 2.03 (d, J = 8.5, 1H), 1.63 (ddd, J_1 = 8.5 Hz, J_2 = 3.6 Hz, J_3 = 1.8 Hz, 1H). ^{13}C NMR ($CDCl_3$): δ_C (ppm) 200.8, 167.1, 153.4, 150.3, 148.9, 139.2, 136.9, 133.2, 129.7, 127.8, 127.6, 127.1, 122.3, 54.4, 52.0, 49.1, 48.8, 48.3, 45.8. MS (ES-API), m/z: calcd for $C_{21}H_{19}NO_3$: 333.14; found: 334.0 [M+H$^+$], 356.0 [M+Na$^+$].

• (3-(4-Chlorophenyl)bicyclo[2.2.1]hept-5-en-2-yl)(pyridin-2-yl)methanone (**3d**)

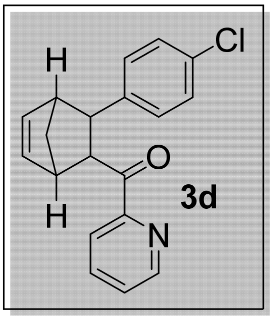

Product **3d** was prepared according to the general methods for the D-A cycloaddition described in the manuscript. Cu(II)-PEIP MOF-catalyzed reaction: 99% conversion, 5:1 mol. ratio of endo:exo. $Cu(OTf)_2$-catalyzed reaction: 99% conversion, 6:1 mol. ratio of endo:exo. Only the endo product was characterized.

Endo: ^{1}H NMR ($CDCl_3$): δ_H (ppm) 8.67 (d, J = 4.6 Hz, 1H), 8.00 (d, J = 7.8 Hz, 1H), 7.83 (dt, J_1 = 7.7 Hz, J_2 = 1.6 Hz, 1H), 7.46 (dd, J_1 = 7.8 Hz, J_2 = 4.9 Hz, 1H), 7.23 (bs, 4H), 6.47 (dd, J_1 = 5.7, J_2 = 3.2 Hz, 1H), 5.83 (dd, J_1 = 5.7, J_2 = 2.8 Hz, 1H), 4.45 (dd, J_1 = 5.3 Hz, J_2 = 3.6 Hz, 1H), 3.54 (bs, 1H), 3.40 (d, J_1 = 5.3 Hz, 1H), 3.05 (bs, 1H), 2.01 (d, J = 8.5 Hz, 1H), 1.62 (ddd, J_1 = 8.5 Hz, J_2 = 3.6 Hz, J_3 = 1.7 Hz, 1H). ^{13}C NMR ($CDCl_3$): δ_C (ppm) 201.1, 153.6, 149.0, 143.3, 139.4, 137.0, 133.1, 131.6, 129.6, 128.6, 127.1, 122.4, 54.6, 49.4, 48.9, 48.3, 45.2. MS (ES-API), m/z: calcd for $C_{19}H_{16}ClNO$: 309.09; found: 310.0 [M+H$^+$], 332.0 [M+Na$^+$].

• (3-(4-Nitrophenyl)bicyclo[2.2.1]hept-5-en-2-yl)(pyridin-2-yl)methanone (**3e**)

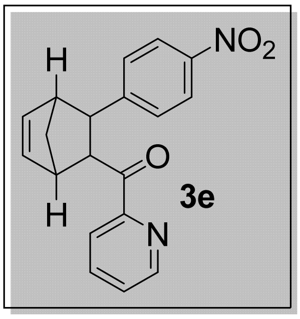

3e

Product **3e** was prepared according to the general methods for the D-A cycloaddition described in the manuscript. Cu(II)-PEIP MOF-catalyzed reaction: 99% conversion, 6.5:1 mol. ratio of endo:exo. Cu(OTf)$_2$-catalyzed reaction: 99% conversion, 6.5:1 mol. ratio of endo:exo. Only the endo product was characterized.

Endo: ^{1}H NMR (CDCl$_3$): δ$_H$ (ppm) 8.66 (ddd, J_1 = 4.8 Hz, J_2 = 1.8 Hz, J_3 = 0.9 Hz, 1H), 8.13 (d, J = 8.8 Hz, 2H), 8.01 (td, J_1 = 7.8 Hz, J_2 = 1.1 Hz, 1H), 7.84 (dt, J_1 = 7.7 Hz, J_2 = 1.7 Hz, 1H), 7.48 (ddd, J_1 = 7.8 Hz, J_2 = 4.8 Hz, J_3 = 1.3 Hz, 1H), 7.45 (d, J = 8.8 Hz, 2H), 6.49 (dd, J_1 = 5.7 Hz, J_2 = 3.2 Hz, 1H), 5.87 (dd, J_1 = 5.7 Hz, J_2 = 2.8 Hz, 1H), 4.47 (dd, J_1 = 5.3 Hz, J_2 = 3.6 Hz, 1H), 3.60 (bs, 1H), 3.54 (d, J = 5.3 Hz, 1H), 3.13 (dt, J_1 = 3.4 Hz, J_2 = 1.7 Hz, 1H), 2.00 (d, J = 8.6 Hz, 1H), 1.67 (ddd, J_1 = 8.6 Hz, J_2 = 3.6 Hz, J_3 = 1.7 Hz, 1H). ^{13}C NMR (CDCl$_3$): δ$_C$ (ppm) 200.5, 153.3, 152.9, 149.0, 146.2, 139.1, 137.1, 133. 5, 128.5, 127.3, 123.7, 122.4, 54.8, 49.0, 48.9, 48.3, 45.8. MS (ES-API), m/z: calcd for $C_{19}H_{16}N_2O_3$: 320.12; found: 321.0 [M+H$^+$], 298.0 [M+Na$^+$].

• (3-(3-Nitrophenyl)bicyclo[2.2.1]hept-5-en-2-yl)(pyridin-2-yl)methanone (**3f**)

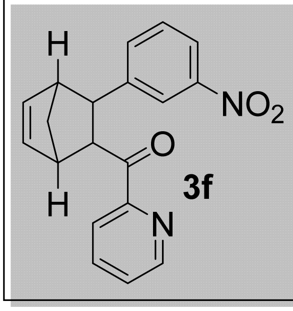

3f

Product **3f** was prepared according to the general methods for the D-A cycloaddition described in the manuscript. Cu(II)-PEIP MOF-catalyzed reaction: 99% conversion, 6.5:1 mol. ratio of endo:exo. Cu(OTf)$_2$-catalyzed reaction: 99% conversion, 10:1 mol. ratio of endo:exo. Only the endo product was characterized.

Endo: ^{1}H NMR (CDCl$_3$): δ$_H$ (ppm) 8.67 (ddd, J_1 = 4.8 Hz, J_2 = 1.8 Hz, J_3 = 0.8 Hz, 1H), 8.19 (app. t, J = 1.8 Hz, 1H), 8.03 (ddd, J_1 = 8.1 Hz, J_2 = 1.8 Hz, J_3 = 0.8 Hz, 1H), 8.01 (dd, J_1 = 7.8 Hz, J_2 = 1.1 Hz, 1H), 7.84 (app. dt, J_1 = 7.7 Hz, J_2 = 1.8 Hz, 1H), 7.62 (ddd, J_1 = 7.7 Hz, J_2 = 1.8 Hz, J_3 = 0.8 Hz, 1H), 7.48 (ddd, J_1 = 7.6 Hz, J_2 = 4.8 Hz, J_3 = 1.1 Hz, 1H), 7.43 (app. t, J = 7.9 Hz, 1H), 6.49 (dd, J_1 = 5.7 Hz, J_2 = 3.2 Hz, 1H), 5.87 (dd, J_1 = 5.7 Hz, J_2 = 2.8 Hz, 1H), 4.49 (dd, J_1 = 5.3 Hz, J_2 = 3.6 Hz, 1H), 3.60 (bs, 1H), 3.53 (d, J = 5.3 Hz, 1H), 3.14 (dt, J_1 = 3.4 Hz, J_2 = 1.6 Hz, 1H), 2.03 (d, J = 8.7 Hz, 1H), 1.67 (ddd, J_1 = 8.7 Hz, J_2 = 3.6 Hz, J_3 = 1.6 Hz, 1H). ^{13}C NMR (CDCl$_3$): δ$_C$ (ppm) 200.5, 153.3, 149.1, 148.6, 147.1, 139.2, 137.2, 134.6, 133.6, 129.4, 127.3, 122.5, 122.2, 121.2, 54.8, 49.3, 48.9, 48.3, 45.5. MS (ES-API), m/z: calcd for $C_{19}H_{16}N_2O_3$: 320.12; found: 321.0 [M+H$^+$], 343.0 [M+Na$^+$].

• *N,N,N*-Trimethyl-4-(3-picolinoylbicyclo[2.2.1]hept-5-en-2-yl)benzenaminium iodide (**3g**)

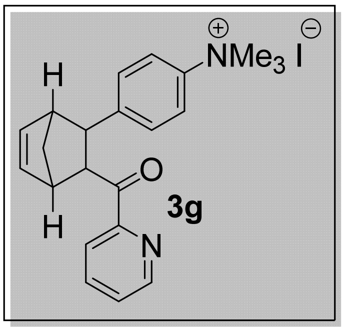

Product **3g** was prepared according to the general methods for the D-A cycloaddition described in the manuscript. Cu(II)-PEIP MOF-catalyzed reaction: 99% conversion, endo only. Cu(OTf)$_2$-catalyzed reaction: 99% conversion, 7:1 mol. ratio of endo:exo. Only the endo product was characterized.

Endo: ^{1}H NMR (CDCl$_3$): δ_H (ppm) 8.66 (ddd, J_1 = 4.8 Hz, J_2 = 1.8 Hz, J_3 = 0.8 Hz, 1H), 7.98 (dd, J_1 = 7.8 Hz, J_2 = 1.2 Hz, 1H), 7.83 (app. dt, J_1 = 7.7 Hz, J_2 = 1.8 Hz, 1H), 7.82 (d, J = 9.0 Hz, 2H), 7.51 (d, J = 9.0 Hz, 2H), 7.47 (ddd, J_1 = 7.6 Hz, J_2 = 4.8 Hz, J_3 = 1.2 Hz, 1H), 6.46 (dd, J_1 = 5.6 Hz, J_2 = 3.2 Hz, 1H), 5.83 (dd, J_1 = 5.6 Hz, J_2 = 2.8 Hz, 1H), 4.43 (dd, J_1 = 5.2 Hz, J_2 = 3.6 Hz, 1H), 3.94 (s, 9H), 3.56 (bs, 1H), 3.45 (d, J = 5.2 Hz, 1H), 3.07 (dt, J_1 = 3.4 Hz, J_2 = 1.7 Hz, 1H), 1.95 (d, J_1 = 8.7 Hz, 1H), 1.63 (ddd, J_1 = 8.7 Hz, J_2 = 3.6 Hz, J_3 = 1.7 Hz, 1H). ^{13}C NMR (CDCl$_3$): δ_C (ppm) 200.6, 153.3, 149.1, 148.3, 145.0, 139.1, 137.1, 133.4, 130.0, 127.3, 122.4, 119.8, 58.0, 54.7, 49.0, 48.9, 48.3, 45.1. MS (ES-API), m/z: calcd for $C_{22}H_{25}N_2O^+$: 333.20; found: 333.0 [M].

• Pyridin-2-yl(3-(*p*-tolyl)bicyclo[2.2.1]hept-5-en-2-yl)methanone (**3h**)

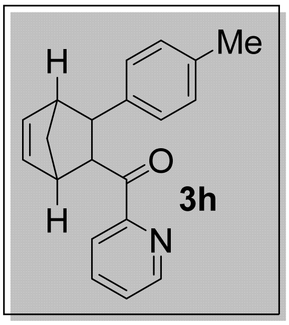

Product **3h** was prepared according to the general methods for the D-A cycloaddition described in the manuscript. Cu(II)-PEIP MOF-catalyzed reaction: 99% conversion, 8:1 mol. ratio of endo:exo. Cu(OTf)$_2$-catalyzed reaction: 57% conversion, 10:1 mol. ratio of endo:exo. Only the endo product was characterized.

Endo: ^{1}H NMR (CDCl$_3$): δ_H (ppm) 8.68 (ddd, J_1 = 4.8 Hz, J_2 =1.8 Hz, J_3=0.9 Hz, 1H), 8.00 (td, J_1 = 7.9 Hz, J_2 =1.0 Hz, 1H), 7.82 (dt, J_1 = 7.7 Hz, J_2 = 1.7 Hz, 1H), 7.45 (ddd, J_1 = 7.6 Hz, J_2 = 4.8 Hz, J_3= 1.3 Hz, 1H), 7.22 (d, J = 8.0 Hz, 2H), 7.09 (d, J = 8.0 Hz, 2H), 6.49 (dd, J_1 = 5.6 Hz, J_2 = 3.2 Hz, 1H), 5.82 (dd, J_1 = 5.6 Hz, J_2 = 2.8 Hz, 1H), 4.53 (dd, J_1 = 5.2 Hz, J_2 = 3.6 Hz, 1H), 3.54 (bs, 1H), 3.41 (d, J = 5.2 Hz, 1H), 3.05 (dt, J_1 = 3.4 Hz, J_2 = 1.7 Hz, 1H), 2.30 (s, 3H), 2.07 (d, J = 8.5 Hz, 1H), 1.60 (ddd, J_1 = 8.5 Hz, J_2 =3.6 Hz, J_3=1.7 Hz, 1H). ^{13}C NMR (CDCl$_3$): δ_C (ppm) 201.3, 153.7, 149.0, 141.7, 139.6, 137.0, 135.4, 132.9, 129.2, 127.7, 127.0, 122.3, 54.3, 49.7, 48.9, 48.3, 45.4, 21.0. MS (ES-API), m/z: calcd for $C_{20}H_{19}NO$: 289.15; found: 290.0 [M+H$^+$], 312.0 [M+Na$^+$].

• (3-(4-Methoxyphenyl)bicyclo[2.2.1]hept-5-en-2-yl)(pyridin-2-yl)methanone (**3i**)

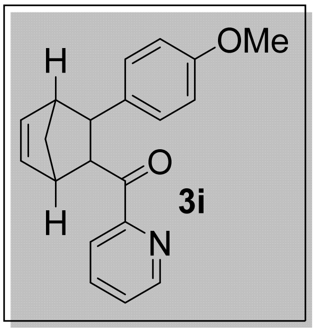

Product **3i** was prepared according to the general methods for the D-A cycloaddition described in the manuscript. Cu(II)-PEIP MOF-catalyzed reaction: 50% conversion, 7:1 mol. ratio of endo:exo. $Cu(OTf)_2$-catalyzed reaction: 54% conversion, 14:1 mol. ratio of endo:exo. Only the endo product was characterized.

Endo: ^{1}H NMR ($CDCl_3$): δ_H (ppm) 8.68 (ddd, J_1 = 4.8 Hz, J_2 = 1.8 Hz, J_3 = 0.8 Hz, 1H), 8.00 (dd, J_1 = 7.9 Hz, J_2 = 1.1 Hz, 1H), 7.81 (dt, J_1 = 7.8 Hz, J_2 = 1.8 Hz, 1H), 7.45 (ddd, J_1 = 7.7 Hz, J_2 = 4.8 Hz, J_3 = 1.1 Hz, 1H), 7.24 (d, J = 8.7 Hz, 2H), 6.82 (d, J = 8.7 Hz, 2H), 6.48 (dd, J_1 = 5.6 Hz, J_2 = 3.2 Hz, 1H), 5.81 (dd, J_1 = 5.6 Hz, J_2 = 2.8 Hz, 1H), 4.49 (dd, J_1 = 5.3 Hz, J_2 = 3.6 Hz, 1H), 3.77 (s, 3H), 3.53 (bs, 1H), 3.39 (d, J = 5.3 Hz, 1H), 3.02 (dt, J_1 = 3.4 Hz, J_2 = 1.7 Hz, 1H), 2.06 (d, J = 8.5 Hz, 1H), 1.60 (ddd, J_1 = 8.5 Hz, J_2 = 3.6 Hz, J_3 = 1.7 Hz, 1H). ^{13}C NMR ($CDCl_3$): δ_C (ppm) 201.4, 157.9, 153.7, 149.0, 139.6, 137.0, 136.8, 132.9, 128.7, 127.0, 122.3, 113.9, 55.4, 54.4, 49.8, 48.8, 48.3, 45.0. MS (ES-API), m/z: calcd for $C_{20}H_{19}NO_2$: 305.14; found: 306.0 [M+H$^+$].

• (3-(4-(Dimethylamino)phenyl)bicyclo[2.2.1]hept-5-en-2-yl)(pyridin-2-yl)methanone (**3j**)

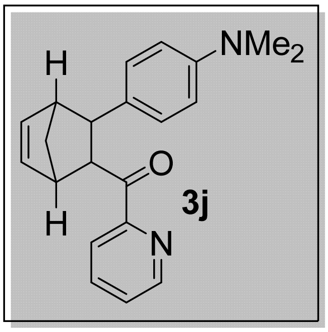

The general methods for the D-A cycloaddition described in the manuscript were applied to synthesize **3j**. Cu(II)-PEIP MOF-catalyzed reaction: ~0% conversion. $Cu(OTf)_2$-catalyzed reaction: ~ 0% conversion.

• Pyridin-2-yl(3-(pyridin-4-yl)bicyclo[2.2.1]hept-5-en-2-yl)methanone (**3k**)

Product **3k** was prepared according to the general methods for the D-A cycloaddition described in the manuscript. Cu(II)-PEIP MOF-catalyzed reaction: 99% conversion, 2:1 mol. ratio of endo:exo. $Cu(OTf)_2$-catalyzed reaction: 80% conversion, 8:1 mol. ratio of endo:exo. Only the endo product was characterized.

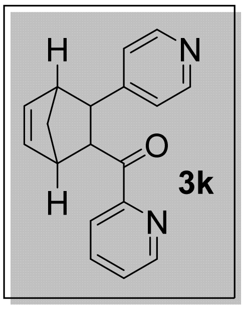

3k

Endo: ^{1}H NMR ($CDCl_3$): δ_H (ppm) 8.66 (ddd, J_1 = 4.5 Hz, J_2 = 1.8 Hz, J_3 = 0.8 Hz, 1H), 8.46 (d, J = 5.3 Hz, 2H), 8.00 (dd, J_1 = 7.9 Hz, J_2 = 0.8 Hz, 1H), 7.83 (ddt, J_1 = 8.5 Hz, J_2 = 7.6 Hz, J_3 = 0.8 Hz, 1H), 7.46 (m, 1H), 7.21 (d, J = 5.3 Hz, 2H), 6.46 (dd, J_1 = 5.7 Hz, J_2 = 3.3 Hz, 1H), 5.84 (dd, J_1 = 5.7 Hz, J_2 = 2.7 Hz, 1H), 4.47 (dd, J_1 = 5.2 Hz, J_2 = 3.6 Hz, 1H), 3.57 (bs, 1H), 3.41 (d, J = 5.2 Hz, 1H), 3.11 (bs, 1H), 1.96 (d, J = 8.6 Hz, 1H), 1.63 (d, J = 8.6 Hz, 1H). ^{13}C NMR ($CDCl_3$): δ_C (ppm) 200.6, 154.1, 153.4, 149.7, 149.0, 139.0, 137.1, 133.5, 127.3, 123.2, 122.4, 54.2, 48.8, 48.8, 48.3, 45.2. MS (ES-API), m/z: calcd for $C_{18}H_{16}N_2O$: 276.13; found: 277.0 [M+H$^+$].

• Pyridin-2-yl(3-(pyridin-2-yl)bicyclo[2.2.1]hept-5-en-2-yl)methanone (**3l**)

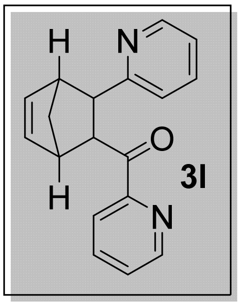

3l

Product **3l** was prepared according to the general methods for the D-A cycloaddition described in the manuscript. Cu(II)-PEIP MOF-catalyzed reaction: 99% conversion, 8:1 mol. ratio of endo:exo. $Cu(OTf)_2$-catalyzed reaction: 50% conversion, endo only. Only the endo product was characterized.

Endo: ^{1}H NMR ($CDCl_3$): δ_H (ppm) 8.66 (d, J = 4.6 Hz, 1H), 8.55 (d, J = 4.6 Hz, 1H), 7.99 (d, J = 7.9 Hz, 1H), 7.80 (dt, J_1 = 7.7 Hz, J_2 = 1.4 Hz, 1H), 7.58 (app. t, J = 7.4 Hz, 1H), 7.43 (dd, J_1 = 7.6 Hz, J_2 = 5.4 Hz, 1H), 7.26 (d, J = 7.8 Hz, 1H), 7.10 (app. t, J = 5.6 Hz, 1H), 6.47 (dd, J_1 = 5.7 Hz, J_2 = 3.2 Hz, 1H), 5.90 (dd, J_1 = 5.7 Hz, J_2 = 2.7 Hz, 1H), 4.94 (d, J = 5.2 Hz, 1H), 3.57 (bs, 1H), 3.51 (d, J = 5.2 Hz, 1H), 3.10 (bs, 1H), 2.29 (d, J = 8.2 Hz, 1H), 1.52 (d, J = 8.2 Hz, 1H). ^{13}C NMR ($CDCl_3$): δ_C (ppm) 201.4, 163.4, 153.8, 149.0, 148.8, 138.9, 136.9, 136.5, 134.1, 126.9, 123.6, 122.3, 121.2, 53.0, 50.4, 48.6, 48.0, 47.7. MS (ES-API), m/z: calcd for $C_{18}H_{16}N_2O$: 276.13; found: 277.0 [M+H$^+$].

• (3-(Furan-2-yl)bicyclo[2.2.1]hept-5-en-2-yl)(pyridin-2-yl)methanone (**3m**)

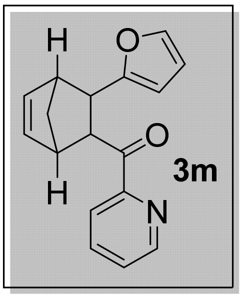

3m

Product **3m** was prepared according to the general methods for the D-A cycloaddition described in the manuscript. Cu(II)-PEIP MOF-catalyzed reaction: 99% conversion, endo only. $Cu(OTf)_2$-catalyzed reaction: 99% conversion, endo only. Only the endo product was characterized.

Endo: ^{1}H NMR ($CDCl_3$): δ_H (ppm) 8.71 (d, J = 4.6 Hz, 1H), 8.00 (d, J = 7.8 Hz, 1H), 7.82 (dt, J_1 = 7.6 Hz, J_2 = 1.6 Hz, 1H), 7.47 (ddd, J_1 = 7.6 Hz, J_2 = 4.8 Hz, J_3 = 1.1 Hz, 1H), 7.30 (d, J = 1.3 Hz, 1H), 6.41 (dd, J_1 = 5.7 Hz, J_2 = 3.2 Hz, 1H), 6.27 (dd, J_1

= 3.3 Hz, J_2 = 1.9 Hz, 1H), 6.10 (d, J = 3.2 Hz, 1H), 5.79 (dd, J_1 = 5.7 Hz, J_2 = 2.8 Hz, 1H), 4.58 (dd, J_1 = 5.2 Hz, J_2 = 3.6 Hz, 1H), 3.53 (bs, 1H), 3.39 (d, J = 5.2 Hz, 1H), 3.07 (bs, 1H), 2.03 (d, J = 8.5 Hz, 1H), 1.56 (ddd, J_1 = 8.5 Hz, J_2 = 3.6 Hz, J_3 = 1.7 Hz, 1H). ^{13}C NMR ($CDCl_3$): δ_C (ppm) 200.4, 158.2, 153.5, 149.0, 141.2, 138.5, 137.1, 132.9, 127.1, 122.4, 110.2, 104.9, 52.3, 49.3, 48.7, 48.3, 39.8. MS (ES-API), m/z: calcd for $C_{17}H_{15}NO_2$: 265.11; found: 265.0 [M].

2. Selected bond lengths and distances in the Cu(II)-PEIP MOF

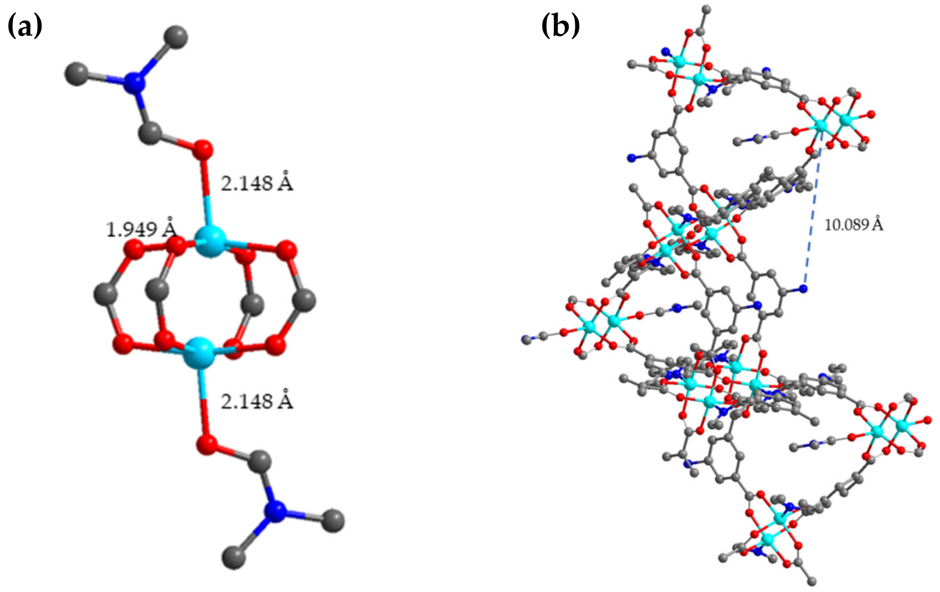

Figure S1. (**a**) Paddle-wheel structure of the Cu(II) coordination sites in the MOF, showing short Cu-O (RCO_2) bonds and long Cu-O (DMF) bonds. (**b**) Distance of a free NH_2 from a Cu(II) catalytic site is comparable to the dimensions of the dienophile.

3. Digestion of Cu(II)-PEIP MOF and ¹H NMR determination of residual DMF

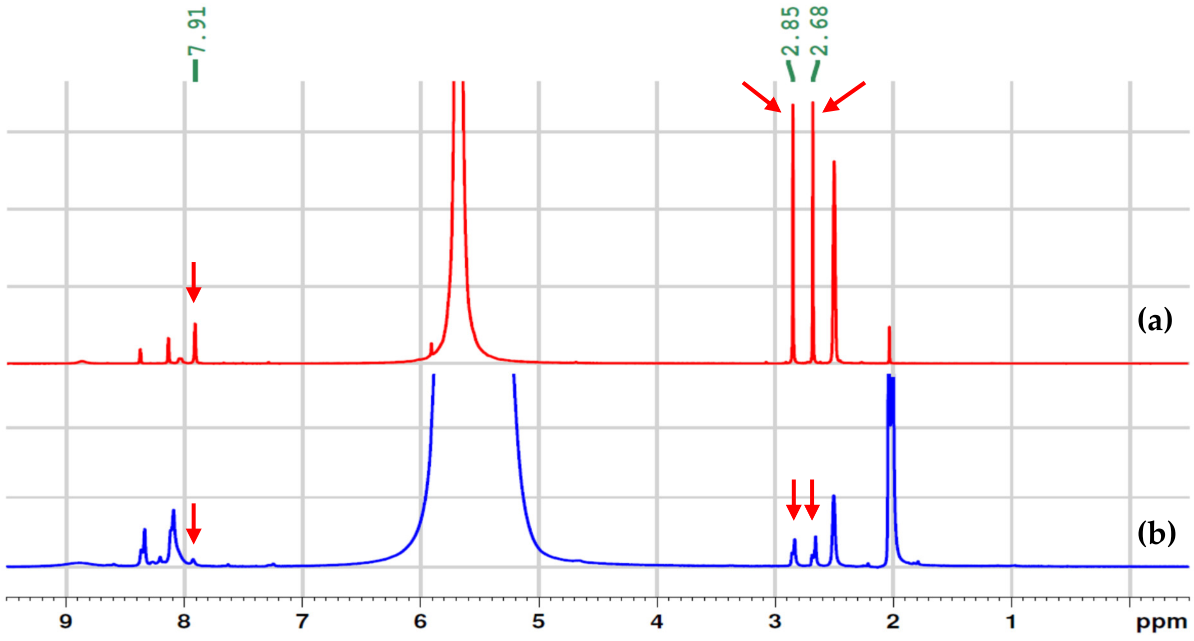

Figure S2. ^{1}H NMR of Cu(II)-PEIP, digested in HCl/DMSO: (**a**) As synthesized, prior to acetone wash (red). DMF, trapped in the MOF's pores, is evident in the spectrum (red arrows). (**b**) After extensive wash with acetone and drying (blue). (Small) residual DMF peaks (red arrows) correspond to DMF coordinated on Cu(II) metal centers.

4. Check of MOF stability under optimized D-A reaction conditions via PXRD

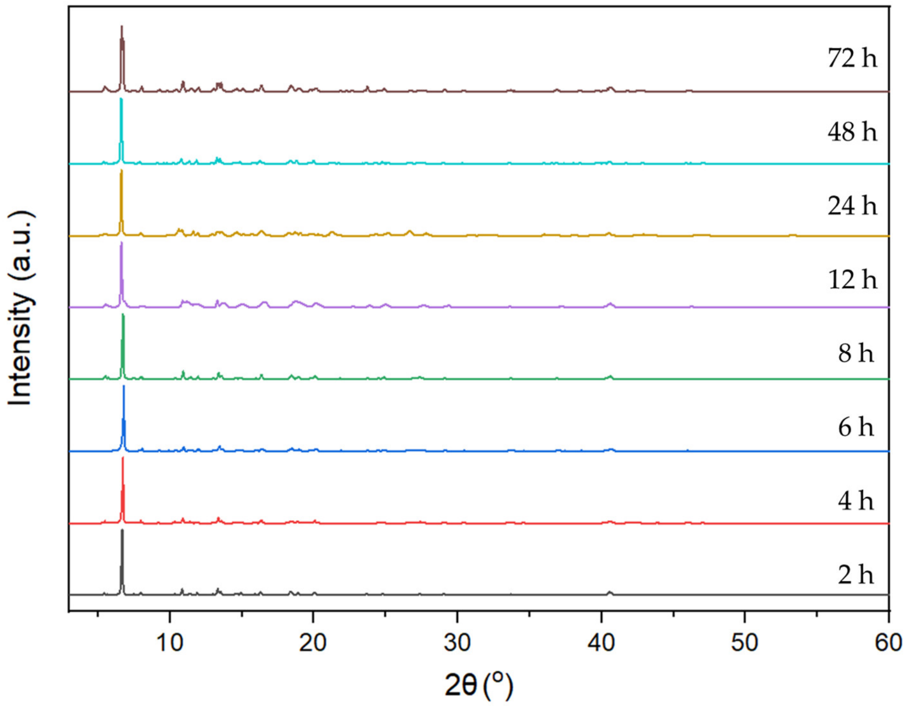

Figure S3. Powder X-ray diffraction patterns of Cu(II)-PEIP catalyst, after exposure to optimized Diels-Alder reaction conditions (H_2O/10% mol SDS), in the absence of substrates, for various times.

5. ^{1}H NMR check of aqueous supernatant for ligand leaching from MOF

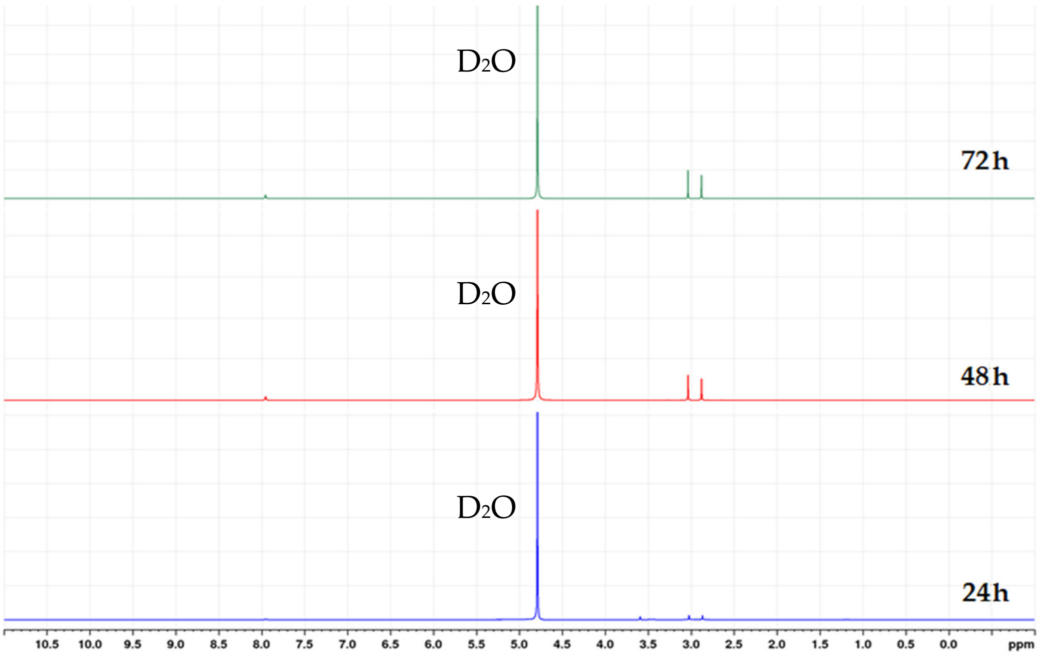

Figure S4. ^{1}H NMR spectra of the supernatant liquid, after submission of the Cu(II)-PEIP catalyst to optimized Diels-Alder reaction conditions (D_2O/10% mol SDS), in the absence of substrates, for various times (24 h, 48 h, 72 h). This study shows that no ligand leaching takes place under these conditions.

6. Comparison of ¹H NMR spectra between different D-A product batches, obtained using MOF catalyst that was exposed to aqueous conditions for variable time

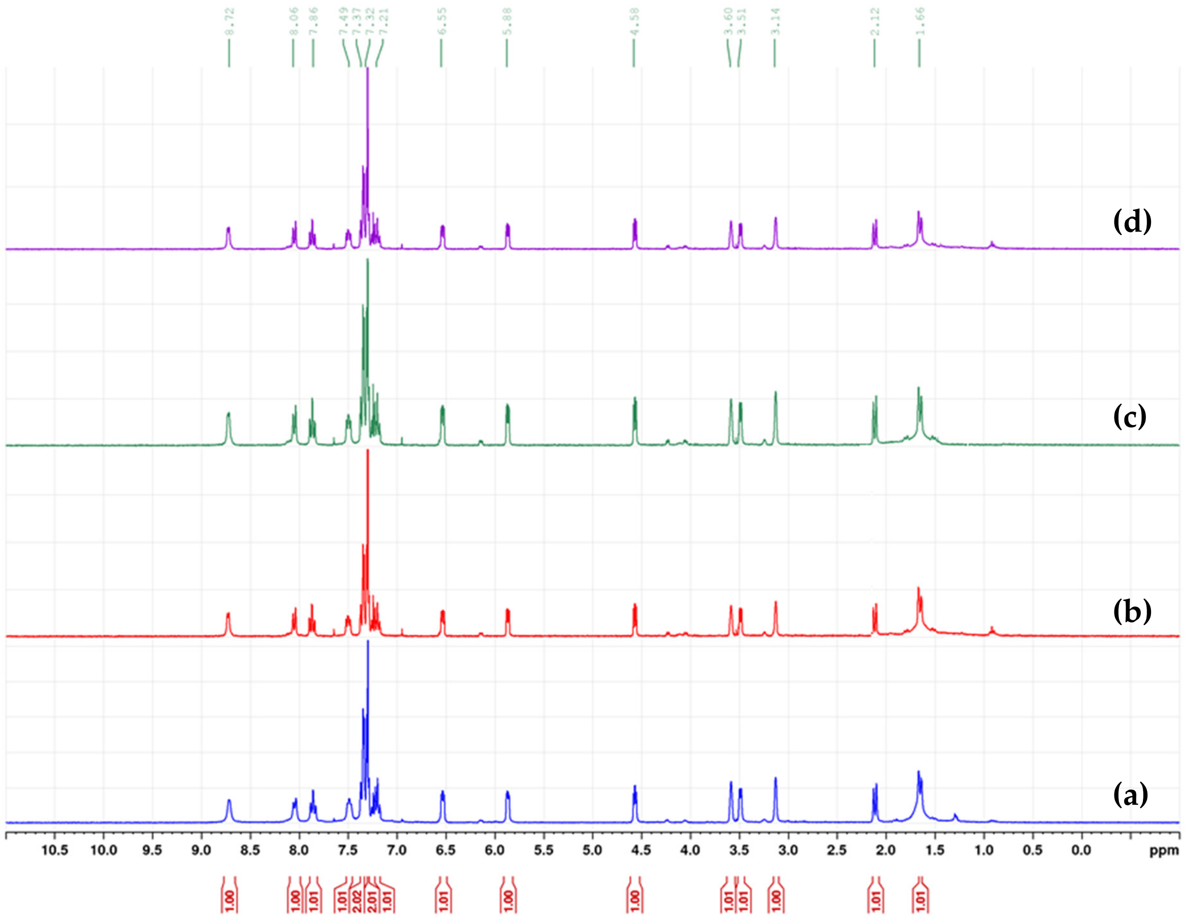

Figure S5. Comparison of ¹H NMR spectra of different batches of product **3a**, obtained from dienophile **1a** and diene **2** via Cu(II)-PEIP-catalyzed Diels-Alder reaction. (**a**)-(**d**) The Cu(II)-PEIP MOF catalyst was exposed to the selected aqueous conditions (D_2O/10% mol SDS), for 0 h, 24 h, 48 h and 72 h, respectively, prior to being used in the D-A reaction. This study indicates the quantitative conversion of **1a** to **3a** in all cases, suggesting that catalyst activity is unaffected by any pre-treatment with D_2O/10% mol SDS.

7. References

S1. Mukherjee, S.; Mondal, S.; Patra, A.; Gonnade, R.G.; Biju, A.T. N-Heterocyclic Carbene-Catalyzed Diastereoselective Synthesis of β-Lactone-Fused Cyclopentanes Using Homoenolate Annulation Reaction. *Chem. Commun.* **2015**, *51*, 9559–9562, doi:10.1039/C5CC02960K.

S2. Basler, S.; Studer, S.; Zou, Y.; Mori, T.; Ota, Y.; Camus, A.; Bunzel, H.A.; Helgeson, R.C.; Houk, K.N.; Jiménez-Osés, G.; Hilvert, D. Efficient Lewis-Acid Catalysis of an Abiological Reaction in a De Novo Protein Scaffold. *Nature Chem.* **2021**, *13*, 231-235, doi:10.1038/s41557-020-00628-4.

S3. Mukkala, V.-M.; Helenius, M.; Hemmilä, I.; Kankare, J.; Takalo, H. Development of Luminescent Europium(III) and Terbium(III) Chelates of 2,2':6',2''-Terpyridine Derivatives for Protein Labeling. *Helvetica Chim. Acta* **1993**, *76*, 1361-1378, doi: 10.1002/hlca.19930760323.

S4. Zhang, Z.; Dong, Y.-W.; Wang, G.-W. Efficient and Clean Aldol Condensation Catalyzed by Sodium Carbonate in Water. *Chem. Lett.* **2003**, *32*, 966-967, doi: 10.1246/cl.2003.966.
